# Comprehensive review on cannabis seed phenolic compounds from extraction to functional applications

**DOI:** 10.1186/s42238-026-00401-3

**Published:** 2026-02-10

**Authors:** Chaymae Benkirane, Rafika El Ati, Malika Abid, Jacques Nkengurutse, Hana Serghini-Caid, Ahmed Elamrani, Farid Mansouri

**Affiliations:** 1 Agricultural Production Improvement, Biotechnology and Environment Laboratory, Faculty of Sciences, Mohammed I University, BP-717, Oujda, 60000 Morocco; 2 Biology Department, Faculty of Sciences, University of Burundi, P.O. Box 2700, Bujumbura, Burundi; 3 Synergy Lab, Higher School of Education and Training, Mohammed I University, BP-410, Oujda, 60000 Morocco

**Keywords:** *Cannabis sativa* L., Seeds, Phenolic compounds, Extraction, Isolation, Biological activities, Industrial applications

## Abstract

**Background:**

*Cannabis sativa* L. seeds have long been regarded as a by-product of cannabis cultivation. Beyond their nutritional value, cannabis seeds are increasingly recognized as a source of specialized phenolic compounds with potential biological relevance. However, research on seed phenolics remains fragmented, often receiving only marginal attention, and a dedicated synthesis focused specifically on these compounds is still lacking.

**Main Body:**

This review provides a focused and up-to-date synthesis of phenolic compounds reported in cannabis seeds, critically examining their chemical diversity, extraction and analytical strategies, and key factors influencing phenolic recovery and composition. Cannabis seeds exhibit a diverse phenolic profile dominated by hydroxycinnamic acid amides (mainly *N-trans-*caffeoyltyramine) and lignanamides (particularly Cannabisins A and B). Their extraction remains largely reliant on conventional solvent-based approaches, with limited adoption of innovative and green technologies, except for ultrasound assisted extraction. Advances in high-resolution analytical tools for phenolic separation and identification enabled comprehensive profiling and a deeper understanding of phenolic diversity. The extractability and final content of phenolic compounds result from a complex interplay between extraction-related parameters, biological variability, and seed processing conditions. Biological activities associated with cannabis seed phenolics include antioxidant, anticancer, neuroprotective, anti-inflammatory, dermo-protective, antibacterial, and metabolic regulatory effects. These activities are discussed with reference to reported mechanisms, such as free radical scavenging, modulation of inflammatory mediators, and inhibition of cancer cell proliferation.

**Conclusion:**

Current evidence highlights cannabis seeds as a promising yet underexploited source of phenolic compounds with potential applications in nutraceutical, pharmaceutical, and dermato-cosmetic fields. Nevertheless, important challenges remain, such as methodological heterogeneity, limited in-vivo and clinical validation, and insufficient data on bioavailability and formulation. Addressing these limitations and gaps through standardized protocols, mechanistic studies, and translational research will be essential to support the effective valorization of cannabis seed phenolics in industrial and health-related applications.

## Background

Cannabis (*Cannabis sativa* L.) is one of the oldest domesticated plants in the world. Its cultivation dates back to the Neolithic period, with archaeological evidence indicating its use 12,000 BCE for fiber production (Friedman and Sirven 2017). Indeed, cannabis is a remarkably versatile plant, with several applications encompassing all its organs: inflorescences, leaves, roots, stems, and even the fruit (Crini et al. 2020). The cannabis fruit is an achene, a type of dry indehiscent fruit, containing a single seed enclosed within a hard protective outer layer. This outer layer, commonly called shell or hull, is covered by a thin pericarp, tightly adhered to the seed (Farag and Kayser 2017). The term “seed” is commonly used in research, even when the whole fruit is analyzed, and will be used throughout this review for consistency. Cannabis seeds, once considered a by-product, are now valued for their exceptional nutritional profile (Montero et al. 2023) and their specialized metabolites, particularly phenolic compounds (Guo et al. 2024).

Cannabis seeds present a high oil content (21 to 37%) rich in polyunsaturated fatty acids (Arango et al. 2024; Lančaričová et al. 2021; Schultz et al. 2020), as well as important fat-soluble compounds, mainly γ-tocopherol and β-sitosterol (Siano et al. 2018; Trovato et al. 2023). Cannabis seeds are also a valuable source of high-quality proteins (21–32%), carbohydrates (25- 43%), organic acids, and essential minerals, supporting their use as functional food ingredients (Alonso-Esteban et al. 2022; Arango et al. 2024; Lančaričová et al. 2021; Schultz et al. 2020; Xu et al. 2021). Several products based on cannabis seeds are already marketed, such as dehulled seeds, seed flour, seed oil, protein seed powder, and seed milk. Additionally, they were successfully incorporated as a fortifying component into foods (pasta, bread, energy bars, and yogurt), or as edible fruit coating (Dabija et al. 2018; Jančíková and Dordevic 2020; Mikulec et al. 2019; Norajit et al. 2011; Teterycz et al. 2021). While these nutritional aspects are well documented, they have often overshadowed seed-specific secondary metabolites. Recently, phenolic compounds are at the forefront of research due to their potential biological activities.

Phenolic compounds are molecules resulting from the plant secondary metabolism. They play a vital role in plants since they are involved in several biological processes of communication and defense against exogenous aggressions (Brglez Mojzer et al. 2016). They are also of great importance for human health thanks to their interesting biological activities, such as their antioxidant, anti-inflammatory, anticancer, and antibacterial properties (Hilal et al. 2024). The main phenolic compounds found in cannabis seeds are hydroxycinnamic acid amides (HCAAs) and lignanamides. HCAAs are derivatives of hydroxycinnamic acids, where the acid is linked to an amine group, forming amide bonds (Roumani et al. 2020). Lignanamides, on the other hand, are amide compounds that are derived from oxidative coupling mechanism with HCAAs as intermediates (Leonard et al. 2020). These two phenolic classes are referred to in the literature as “phenylpropanamides” or “phenylpropionamides,” reflecting their biosynthetic origin within the phenylpropanoid pathway. In this review, the term “phenylpropionamides” is used throughout for consistency.

Growing evidence highlights phenylpropionamides as major contributors to the therapeutic properties of cannabis seeds, and research on these compounds has intensified in recent year. Additionally, methodological advances have markedly enhanced the characterization of phenolic compounds in cannabis seeds. The development of optimized extraction strategies, combined with high-resolution analytical tools and metabolomic approaches, has enabled more comprehensive profiling and improved understanding of phenolic diversity. Despite these advances, available data remain scattered across studies.

Numerous literature reviews have explored the phytochemistry and bioactive compounds of the cannabis plant in recent years. However, most studies have focused primarily on cannabinoids, the emblematic compounds of the plant (Pattnaik et al. 2022; Pravat et al. 2023; Valizadehderakhshan et al. 2021), or take a broader approach combining different classes of bioactive compounds and plant organs (Dalli et al. 2023; Guo et al. 2024; Hourfane et al. 2023; Isidore et al. 2021; Kamle et al. 2024; Liu et al. 2022; Martinez et al. 2023; Pollastro et al. 2018). Consequently, phenolic compounds in cannabis seeds are often addressed only marginally, and a dedicated, up-to-date synthesis focused specifically on seed phenolics is still lacking. This gap is particularly evident for phenylpropionamides (HCCAs and lignanamides), whose occurrence, extraction, and biological relevance warrant a consolidated analysis.

**Fig. 1:**
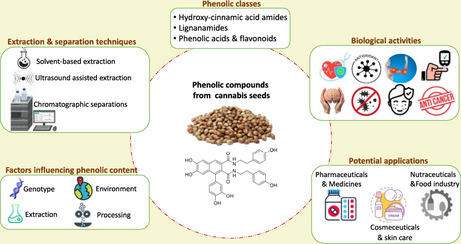
Graphical overview of the key objectives of the present review

The present narrative review aims to provide a comprehensive and seed-specific overview of phenolic compounds in *Cannabis sativa* seeds and their related by-products (cake, meal, hulls, flour). It highlights the different phenolic classes, explores the various extraction, separation, and identification techniques used, and discusses the key factors influencing the phenolic content. In addition, it outlines the biological activities of these compounds and their potential applications in different fields. Figure 1 presents a graphical presentation of the main objectives of this review. By synthesizing recent advances and identifying current limitations, this review seeks to support future research and industrial applications of cannabis seed phenolics.

## Methodology of research

This review was conducted following a structured literature search strategy to ensure a comprehensive and up-to-date synthesis of the available knowledge on phenolic compounds in *Cannabis sativa* seeds. The search was carried out using electronic databases including Scopus, Web of Science, ScienceDirect, PubMed, and Google Scholar. The keywords and combinations used were: *“Cannabis sativa”*, *“cannabis seeds”*, *“phenolic compounds”*, *“polyphenols”*, *“Hydroxycinnamic acid amides”, “lignanamides”*, *“phenolic acids”*, *“flavonoids”,* and *“biological activity”*. The search covered publications up to 2025, with no strict lower time limit, in order to capture both foundational and recent studies. Articles were selected based on their relevance to the objectives of the review. Inclusion criteria comprised peer-reviewed original research articles published in English and focusing on the chemical composition, extraction, characterization, and/or biological activities of phenolic compounds in cannabis seeds. In this context, cannabis seeds refer to seeds from *Cannabis sativa* plants encompassing both low-Δ⁹-tetrahydrocannabinol (THC) varieties (commonly termed hemp) and high-THC varieties, with THC denoting the principal psychoactive constituent used to distinguish cannabis types in the literature. Non-peer-reviewed material and studies dealing exclusively with cannabinoids, agronomic performance, or non-seed plant organs were excluded. After title and abstract screening, duplicate removal, and full-text assessment of eligible articles, relevant data were extracted to synthesize current knowledge and identify research gaps.

## Phenolic compounds in cannabis seeds

The phenolic compounds in cannabis seeds are classified into four major groups. Cannabis seeds are primarily rich in hydroxycinnamic acid amides and lignanamides, followed by phenolic acids and flavonoids, which are present in smaller quantities. Cannabinoids, the iconic compounds of the cannabis plant, are terpenophenolics and can be classified as phenolic compounds. They are primarily secreted in the trichomes and are generally concentrated in the female inflorescences and leaves. They are found in lesser quantities in the stems, and are absent in the seeds and roots. However, several authors have detected the presence of certain cannabinoids in cannabis seeds (Arango et al. 2024; Benkirane et al. 2022; Ferri et al. 2024; Hwang et al. 2025; Song et al. 2022; Yang et al. 2017). This is probably due to exogenous contaminations from small stem/leaf fragments or from some trichomes that stick at the external surface of seeds during harvesting.

Much interest has been devoted to cannabinoids since their initial discovery in the late nineteenth century (Wood et al. 1899). In contrast, research on the major phenolic compounds of cannabis seeds began only in the 1990 s, with the work of Yamamoto et al. (1991) and Sakakibara et al. (1991), who identified the first HCAAs and lignanamides in cannabis seeds. Hydroxycinnamic acid amides, also called phenolamides, or phenylamides are formed by the conjugation of hydroxycinnamic acids (such as caffeic, ferulic, and *p*-coumaric acids) with amines (such as tyramine, octopamine, and spermidine) forming amide bonds. Lignanamides are characterized by a lignan-like core linked to an amide group, often derived from HCAAs via oxidative coupling. Lignanamides have often been named based on the species in which the compound was first discovered; for example, cannabisins from *Cannabis*, lyciumamides from *Lycium* and limoniumins from *Limonium*. Recently, van Zadelhoff et al. (2021) proposed a new systematic nomenclature in which precursors and bond types are unambiguously indicated.

Yamamoto et al. (1991) reported for the first time the presence of two phenolamides (*N-trans*-feruloyltyramine and *N*-*p*-coumaroyltyramine) in cannabis seeds. These two molecules were also detected and isolated by Sakakibara et al. (1991), along with *N-trans*-caffeoyltyramine and grossamide which were probably first reported in cannabis seeds. Additionally, Sakakibara isolated several new lignanamides, namely cannabisins A, B, C, D, E, F and G (Sakakibara et al. 1995, 1992, 1991). Then, cannabisin H and grossamide K were first isolated from the bark of kenaf (*Hibiscus cannabinus*) (Seca et al. 2001) and subsequently identified in cannabis seeds as well. Research has been continued intensively during the last decade (2014–2024) and new compounds have been characterized. Lesma et al. (2014) identified four novel lignanamides (3,3'-demethyl-cannabisin G, 3-demethyl-cannabisin G, 3,3'-demethyl-grossamide, and a cannabisin A derivative). Similarly, Yan et al. (2015) characterized 4 novel lignanamides (cannabisin M, N, O and 3,3'-demethyl-heliotropamide), as well as 10 known lignanamides, 4 of which were identified from cannabis seeds for the first time. Since then, other novel molecules have been isolated such as cannabisin I (Bourjot et al. 2016) and cannabisin Q along with other phenolamides reported in cannabis for the first time; *e.g*. *N-trans*-caffeoyloctopamine and *N-trans*-coumaroyloctopamine (Zhou et al. 2018b). In addition, two enantiomers of nor-lignanamides have been isolated from cannabis seeds, namely (±)-Sativamides A and B (Zhu et al. 2018). Nor-lignanamides are lignanamide derivatives that have lost one or more carbon atoms. Recently, a study successfully identified 54 phenylpropionamide compounds, including 14 novel ones, which were designated as cannabisin I–XIV (Zhang et al. 2024). In the same study, the authors identified other lignanamides and phenolamides never detected before in cannabis seeds, such as lyciumamides N, B, and C, limoniumins A and F, thoreliaide B, and (7'R)- *N-trans*-cinnamoyloctopamine.

As for phenolic acids, cannabis seeds contain gallic acid, *p*-hydroxybenzoic acid, protocatechuic acid and lower levels of syringic, caffeic, *p*-coumaric, ferulic, *trans*-cinnamic, vanillic, sinapic and isoferulic acids (Alonso-Esteban et al. 2022; Babiker et al. 2021; Frazzini et al. 2024; Irakli et al. 2019; Liang et al. 2018; Menga et al. 2022; Teh et al. 2014a). The flavonoids reported in a recurring manner in cannabis seeds are catechin, naringin, quercetin, and rutin (Babiker et al. 2021; Haddou et al. 2023; Liang et al. 2018). Other researchers have also found naringenin and epicatechin (Menga et al. 2022), luteolin (Teh et al. 2014a), apigenin (Aloo et al. 2023), as well as some glycosylated derivatives of vitexin (glucosylvitexin, vitexin-2-O-rhamnoside) (Frazzini et al. 2024), and other flavonol glycosides (Quercetin pentoside, Kaempferol pentoside, Quercetin-7-O-acetyldeoxyhexose) (Nigro et al. 2020). Table 1 presents the phenolic compounds identified in cannabis seeds, organized by phenolic class and illustrated with their chemical structures.

**Table 1:**
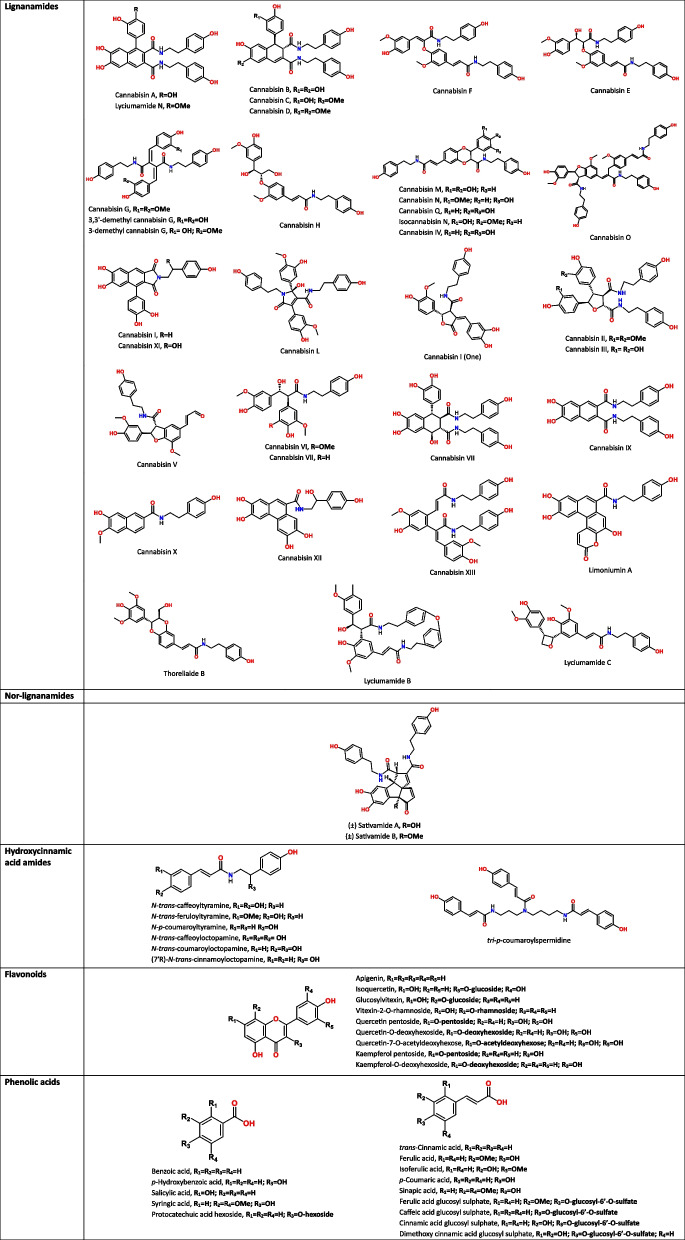
Chemical structures of phenolic compounds identified in cannabis seeds, classified by phenolic classes

**Fig. 2:**
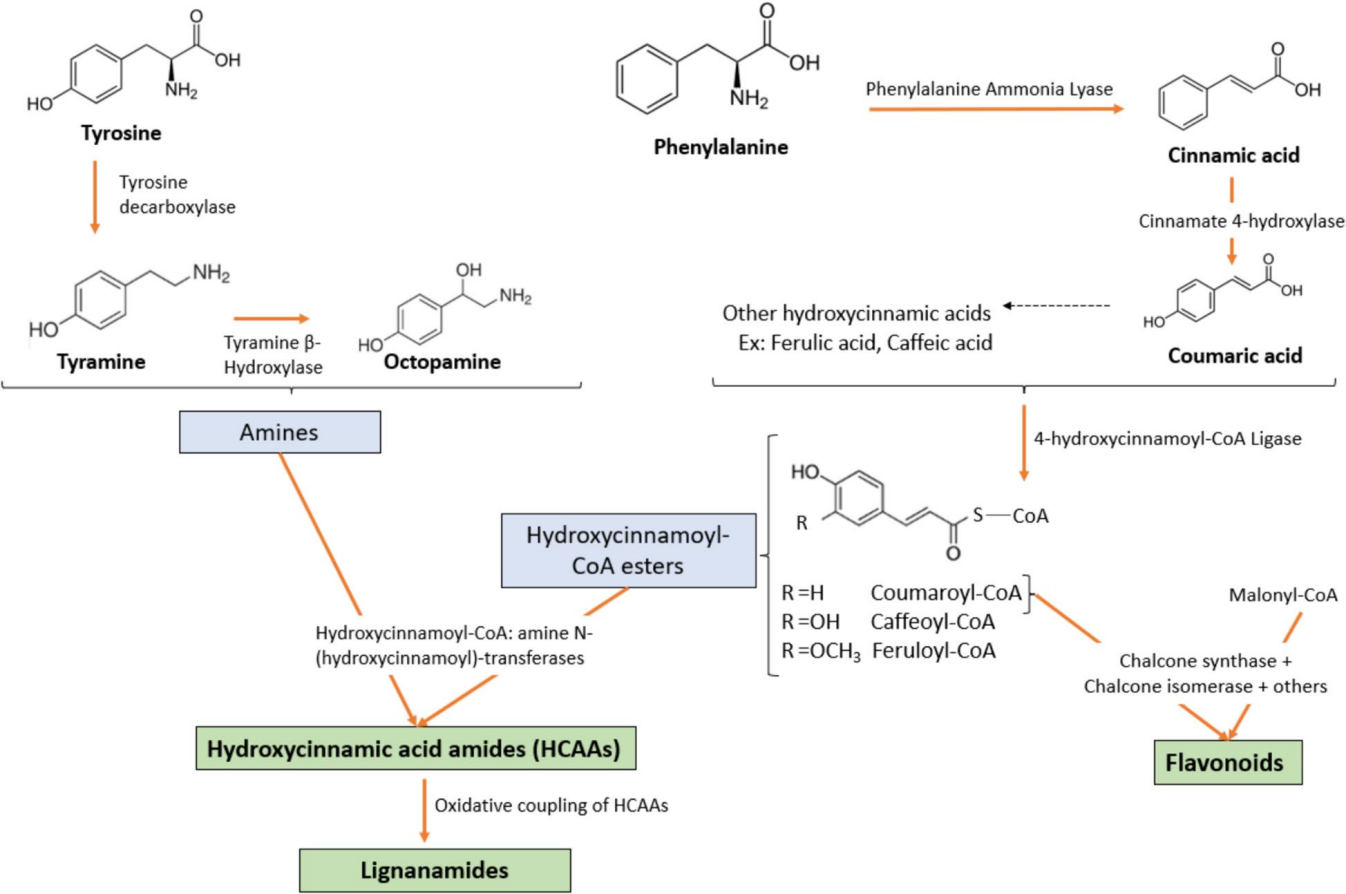
Proposed biosynthesis pathway of phenolic compounds in cannabis seeds

The biosynthesis of phenolic compounds in *Cannabis sativa* seeds is primarily driven by the phenylpropanoid pathway. This pathway begins with the deamination of L-phenylalanine by phenylalanine ammonia-lyase (PAL), yielding cinnamic acid. This latter is then hydroxylated by cinnamate 4-hydroxylase (C4H) to form p-coumaric acid, which undergoes further hydroxylation and methylation through the action of enzymes such as *p*-coumarate 3-hydroxylase (C3H) and caffeic acid O-methyltransferase (COMT), leading to the production of caffeic acid and ferulic acid. These hydroxycinnamic acids are subsequently converted into their corresponding CoA esters by 4-hydroxycinnamoyl-CoA ligase (4CL), serving as key intermediates for the synthesis of various phenolic derivatives (Leonard et al. 2020). The biosynthesis of flavonoids initiates when *p*-coumaroyl-CoA condenses with malonyl-CoA, catalyzed by chalcone synthase (CHS). The resulting chalcone undergoes isomerization and structural modifications via a series of enzymes to yield diverse flavonoid structures (Liu et al. 2021). In parallel, hydroxycinnamic acid amides (HCAAs) are synthesized through a condensation reaction between hydroxycinnamic acid-CoA esters and aliphatic or aromatic amines, a reaction catalyzed by hydroxycinnamoyl-CoA: amine N-hydroxycinnamoyl transferases (Flores-Sanchez and Verpoorte 2008). These amine donors, such as tyramine and octopamine, are derived from the amino acid metabolism (*e.g*., tyrosine). The resulting HCAAs can undergo oxidative coupling, mediated by oxidative enzymes, contributing to the structural diversity of phenolic compounds (Leonard et al. 2020). A general scheme of phenolic biosynthesis in cannabis seeds is illustrated in Fig. 2.

Tables 2 and 3 present the main results regarding phenolic compounds extracted from cannabis seeds and their by-products, with studies organized according to the extraction approach employed. Table 2 compiles results obtained using conventional extraction techniques, whereas Table 3 focuses on unconventional ultrasound assisted extraction. Most studies utilized spectrophotometric assays, particularly the Folin-Ciocalteu method, for determining phenolic content. This method measures the overall reducing capacity of the extract and determines the Total Phenolic Content (TPC) rather than individual phenolic compounds. Reported TPC values varied widely across studies and should be interpreted with caution. Differences in extraction procedures, expression basis (seed material *vs*. extract), and calibration standards (gallic acid or alternative phenolic references) substantially limit cross-study comparability. For indicative purposes, TPC values expressed on a seed basis span a broad range, approximately 90—5000 mg GAE/100 g seeds (Moccia et al. 2020; Song et al. 2022; Taaifi et al. 2021; Teh et al. 2014a; Teh and Birch 2014; Vonapartis et al. 2015), and should be regarded as context-dependent rather than directly comparable. Detailed datasets are provided in Tables 2 and 3.

**Table 2:** Studies using conventional solvent extraction of phenolic compounds from cannabis seeds and their by-products

Plant material (Variety/region)	Extraction conditions	Analytical techniques (isolation/identification)	Main results		Reference
			**Phenolic content**	**Biological activity**	
Seeds (collected from Bama, China)	Defatting with PE Percolation with 75% ethanol three times (3 days each)	• Liquid–liquid partition • RP-LC • RP-MPLC • Sephadex LH-20 CC • MCI gel CC • silica gel CC • HPLC • NMR, HR-MS, UV, IR	• Isolation of 4 new lignanamides (cannabisin M, cannabisin N, cannabisin O, and 3,3′-demethyl-heliotropamide) • Isolation of 10 known lignanamides (4 identified for the first time in hemp seed)	• Good antioxidant activity for several compounds (e.g. IC_50_(DPPH) = 23.9µM for Cannabisin D Vs. Quercetin 25.5µM) • Neuroprotective activity: *N-trans*-caffeoyltyramin, 3,3′-demethyl-heliotropamide, and 3,3′- demethyl-grossamide inhibited acetylcholinesterase (83.28, 62.84, and 87.37% respectively)	(Yan et al. 2015)
Seeds (collected from Bama, China)	Defatting of 3 kg with PE (25 L) (1 h, 3 times) Extraction with 70% ethanol under reflux (3 times, 25 L × 2 h)	• D101 macroporous adsorption resin • HPLC − DAD	• Extraction of a fraction rich in phenylpropionamides • Identification of 14 compounds, with a total content of 233.52 ± 2.50 μg/mg extract	• Anti-neuroinflammatory activity using a lipopolysaccharide (LPS)-induced mouse model: improvements in learning and spatial memory + reduction in pro-inflammatory cytokines + attenuation in neuronal damage in the hippocampus	(Zhou et al. 2018a)
Seeds (collected from Bama, China)	Defatting of 10.7 kg with hexane (2times, 30L, 60 h) Extraction with 95% ethanol under reflux (3 times, 50L, 2 h)	• AB-8 macroporous adsorption resin • Sephadex LH-20 CC • RP- MPLC • HSCCC • silica gel CC • TLC • semi-preparative HPLC	• Isolation of one novel lignanamide (Cannabisin Q), and one novel coumaroylamino glucoside derivative • Isolation of other known phenolics	• Anti-neuroinflammatory activity: Significant inhibitory effects on pro-inflammatory cytokine release from LPS-induced BV2 microglia cells (especially the coumaroyl amino glucoside derivative)	(Zhou et al. 2018b)
Seeds (collected from Bama, China)	Defatting with PE Extraction by reflux with 50% ethanol, 1:25 (g/mL), 45 min	• D101 macroporous resin • Spectro-photometric analysis • HPLC–DAD • HPLC–MS	• TPC: 238.03 μg Caffeic acid equivalent/mg extract • CT: 20.16 μg/mg • CA: 32.16 μg/mg • CB: 214.35 μg/mg	• Neuroprotective activity: using an APP/PS1 transgenic mouse model of Alzheimer’s disease	(Yang et al., 2024)
Seeds of Futura 75	1 g + 10mL 80% ethanol, shaking 3 h, 4 °C in dark	• Spectro-photometric Analysis • HR-MS	• TPC (2.33 mg GAE/g DW), TFC (2.93 mg QE/g DW), and flavonol content (0.85 QE/g DW) • Detection of some cannabinoids and caffeoyltyramine, cannabisin A, B, and C	• Antimutagenic activity: reduced DNA mutations in yeast cells • Antioxidant activity in vitro (DPPH: 40% inhibition, ORAC: 127 μmol TE/g FW) • Antioxidant activity ex vivo: based on hemolysis test on human red blood cells (52% hemolysis inhibition) and CAA-RBC test (CAA = 82) • Antimicrobial activity: Selective activity against pathogenic strains and no inhibitory effects on probiotic strains • Antibiofilm activity: inhibition of the biofilm producer *S. aureus* ATCC35556 strain	(Frassinetti et al. 2020, 2018)
Hulled seeds	Defatting of 5 g with hexane Defatted seeds + 20 mL (1:1) Water: acetonitrile (2min agitation) + QuEChERS salt (2min agitation) Pellet + 10mL MeOH (2 min agitation)	• Spectro-photometric Analysis • HPLC-Q-TOF–MS/MS	• Presence of phenolic acids (4-hydroxybenzoic acid, *p*-coumaric acid, isoferulic acid), and flavonoids (Glucosylvitexin, and Vitexin-2-O-rhamnoside) • TPC (251.8 mg TEA/g), and TFC (71.16 mg CE/g)	• Antioxidant activity (ABTS assay): 60.00% inhibition at 1:2 dilution • Antimicrobial activity against *Escherichia coli* O138: Inhibition of 87% at a concentration of 10 mg/mL	(Frazzini et al. 2024)
Seed hulls	1000 g + 50L Ethanol (10 days)	• Fractionation (hexane, chloroform, ethyl acetate, Water) • normal phase column • RP- column chromatography • ^1^H-/^13^C NMR and mass spectrometry	• Isolation of 9 phenolic compounds: *N-trans*-caffeoyloctopamine, *N-trans*-coumaroyltyramine, *N-trans*-feruloyltyramine, cannabisin F, *N-trans-*caffeoyltyramine, cannabisin A, cannabisin B, grossamide, and cannabisin I	• Anti-tyrosinase activity: - Cannabisins A and B had more potent activity than kojic acid - Cannabisin A decreased the melanin content and tyrosinase activity in B16F10 melanoma cells • Inhibition of soluble epoxide hydrolase: Cannabisins I, A, B, *N-trans*-caffeoyltyramine, and grossamide IC_50_ = 2.7—18.3 μM	(Kim et al. 2024) (Kim et al. 2023)
Seeds (commercially acquired in the province of Seville, Spain)	Defatting with hexane Extraction1 with Acetone, then 50% ethanol (F03) Extraction 2 with 75% Ethanol	• Solvent Partitioning - Extract1: ethanol 96% (F02) - Extract2: (ethyl acetate (F05) and butanol (F06) • Spectro-photometric Analysis • UHPLC-HRMS/MS	• Identification of phenolic acids, flavonoids, and phenolic amides • Most efficient extraction: Ethanol (75%) + ethyl acetate (F05) • The major compound was CT (6.36 mg/g in F05) • TPC and TFC in F05 (5.67 mg GAE/100 mg and 1.76 mg QE/100 mg, respectively)	• Antioxidant activity: particularly F05 (IC_50_ = 211 and 34 µg mL^−1^ in DPPH and ABTS assays) • Anti-inflammatory activity: (particularly F05) by suppressing key inflammatory mediators and shifting monocyte populations toward an anti-inflammatory phenotype	(Rea Martinez et al. 2020)
• Whole seeds of 8 varieties: ‘Bialobrzeskie’ ‘Carmagnola’ ‘Fedora 17’ ‘Felina 32’ ‘KC Dora’ ‘Kompolti’ ‘Santhica 27’ ‘Tiborszallasi’ • 8 dehulled commercial samples	(20 mesh) 1g/50mL of Hydro-methanol, Stirring (25 °C, 150rpm, 1 h) repeated twice	• UPLC system coupled with a DAD and ESI–MS	• Identification of two phenolic acids: ferulic acid-hexoside (0.266 and 0. 619 mg/g) and syringic acid (0.29 to 0.72 mg/g extract)	• Antioxidant activity**:** higher in whole seeds than dehulled seeds, especially in the TBARS assay • Antibacterial activity: notably against *Bacillus cereus* and *Enterococcus faecalis* • Antifungal activity: not remarkable, except for ‘KC Dora’ variety against *Penicillium* (*P. ochrochloron and P. funiculosum*) • Cytotoxic activity, particularly against non-small cell lung cancer (NCI-H460)	(Alonso-Esteban et al., 2022)
Whole seeds of Cheongsam cultivar from Korea	5 g, 70% ethanol, 1:20(w/v), shaking, 50 °C for 1 h, repeated twice	• Spectro-photometric Analysis • UHPLC-Q-TOF–MS/MS	• TPC (379.88 mg FAE/100g), TFC (107.50 mg CE/100g), tannins (215.50 mg CE/100g), and saponins (123.36 mg SSE/100g) • Major polyphenols: Quercetin, apigenin, and rutin • Minor polyphenols: ferulic acid, caffeic acid, and *p*-coumaric acid	• in vitro antioxidant activity: DPPH, ABTS, and FRAP • Anti-obesity and anti-diabetic activities: 75.67% and 76.14% inhibition of lipase and alpha-glucosidase (respectively) at 1 mg/mL • Anti- inflammatory activity: 61.20% inhibition of lipid denaturation	(Aloo et al. 2023)
Seeds (Ketama, Morocco)	60 g + 100mL solvent (Hexane, Ethanol, Dichloromethane Water), stirring (2h for hexane, 24 h for other solvents)	• Spectro-photometric Analysis • HPLC–DAD	• TPC: up to 130 µg GAE/mg extract for the ethanolic extract • Detection of some phenolic acids and flavonoids (*e.g.* naringin, rutin, cinnamic acid), depending on the solvent	Not studied	(Haddou et al. 2023)
Dehulled seeds of ordinary and cheongsam varieties From 6 regions of Korea	(20 mesh) 5 g + 50mL of 70%methanol, maceration 24h	• Spectro-photometric Analysis	• TPC (281.63- 463.31 mg GAE/kg) and TFC (17.23- 35.88 mg CE/kg), depending on variety and region	• Antioxidant activity: DPPH, ABTS, and FRAP	(Hwang et al., 2025)
Hempseed cakes (Fedora 17, France)	Defatting (7.5 kg) with PE (5 × 5L) Extraction with methylene chloride (5 × 5L), then methanol (5 × 5L) at room temperature	• silica gel CC • TLC • sephadex LH-20 column • semipreparative C18 column • HPLC • NMR • HR-MS	• Isolation of 10 compounds (cannabisins A, B, C, F, I, M, 3,3′-demethylgrossamide, grossamide, *N-trans*-caffeoyltyramine, and *N-trans*-caffeoyloctopamine)	• Antioxidant activity (ORAC test): Particularly Cannabisin B, Cannabisin F and *N-trans*-Caffeoyltyramine (9.8, 8.9, 8.9 µmol of TE/µmol of pure compounds) • Anti-arginase activity: Particularly *N-trans*-Caffeoyltyramine IC_50_ = 20.9µM	(Bourjot et al. 2016)
Hemp seed oil Cake (CRS1 Cv., Tasmania)	**Free phenolics**: 35mL of 80%methanol + 4 g of cake, shaking 2 h at RT **Bound phenolics**: Boiling the residue with 30mL 2M HCl (100 °C) for 1 h + 50mL ethyl acetate	• Spectro-photometric Analysis • HPLC–DAD-ESI-QTOF-MS/MS	• TPC (0.385- 0.906 mg GAE/g sample) depending on extrusion parameters • 26 phenylpropionamides • CT: 9.9- 85.77 µg/g sample • CA: 2.77- 7.50 µg CTE/g sample • CB: 3.28- 15.80 µg CTE/g sample	At 1 mg/mL sample: • Antioxidant activity: DPPH and ABTS • Antidiabetic activity: 24–29% inhibition of alpha-glucosidase • Neuroprotective activity: 30–40% inhibition of acetylcholinesterase	(Leonard et al., 2021a)
Hemp seed hull (CRS1 Cv., Tasmania)			• 26 phenylpropionamides • TPC: 2.85 mg GAE/g raw sample • 25–78% increase in total phenylpropionamide content due to extrusion • CT: 128.12–242.46 µg/g sample • CA: 10.59–27.41 µg CTE/g sample • CB: 18.22–63.80 µg CTE/g sample	At 0.2 mg/mL sample: • Antioxidant activity: DPPH and ABTS • Antidiabetic activity: 41–57% inhibition of alpha-glucosidase • Neuroprotective activity: 63–68% inhibition of acetylcholinesterase	(Leonard et al., 2021b)
Seeds of C. sativa L.: Carmagnola, Futura 75, and Felina 32 + 2 sites in Italy	Defatting 40 g with heptane (400mL) Defatted seeds + 200mL 80% methanol, stirring 3h	• Silica gel CC • Sephadex Chromatography • HPLC–MS • NMR spectroscopy	• Identification of six known and four new lignanamides (3,3’-demethyl-cannabisin G; 3-demethyl-cannabisin G; 3,3’-demethyl-grossamide, cannabisin A derivative)	Not studied	(Lesma et al. 2014)
Defatted hemp seeds	6 g + 100mL solvent (Hexane, Methanol, ethanol, acetone, methanol 80%, acetone 80%, methanol:acetone: water (MAW; 7:7:6) (1h, RT, 1000rpm)	• Spectro-photometric Analysis • HPLC–DAD	• TPC (167–733 mg GAE/100 g FW), TFC (0.23–27 mg LUE/100 g FW), depending on solvents • MAW had the highest phenolic and flavonoid contents • Identification of Quercetine, Caffeic acid, luteoline	• Antioxidant activity (DPPH/FRAP): extracts with MAW had the highest reducing power and % inhibition DPPH	(Teh et al. 2014a, b)
Whole seeds from seven hemp varieties	100 mg + 1.2mL 70% methanol, vortex 30 s every 30 min (6times)	• UHPLC-QQQ-MS/MS	• Identification of 1,001 metabolites, including 201 flavonoids, 86 alkaloids, and 149 phenolic acids	• Antioxidant activity: DPPH (69.71–91.91% inhibition), FRAP (17.39–32.53 μmol Fe^2+^/g)	(Ning et al. 2022)
Seed shell	2 g + 80mL 70% methanol, stirring 200 rpm, 25 °C, 10-300min	• Spectro-photometric Analysis • HPLC–DAD	Depending on particle size: • CT (12.81- 15.81 mg/g extract) • CA (39.67- 48.68 mg/g extract) • CB (46.11 −55.75 mg/g extract) • TPC: 420.64- 435.53 mg GAE/g extract • TFC: 376.75- 416.47 mg RE/g extract	• In vitro Antioxidant activity: DPPH and ABTS with IC_50_ values of 36.13–44.2 μg/mL and 32.30–39.45 μg/mL, respectively • Cellular Antioxidant activity in HUVEC cells: reducing reactive oxygen species production, lipid peroxidation, and LDH leakage, while increasing SOD activity	(Xu et al. 2024)
Seeds (unroasted and roasted) Turkey	1 g + 10mL ethanol: water (80:20) in rinsing water-bath for 3 h at 4 ◦C,	• Spectro-photometric Analysis • HPLC–PDA	• TPC and TFC of unroasted seeds were 16.67 mg GAE/100 g FW and 29.0 mg CE/100 g FW • Identification of phenolic acids and flavonoids • Phenolics increase with roasting time (14 min optimal)	• Antioxidant activity: 18.37% DPPH scavenging of the unroasted seeds	(Babiker et al. 2021)
Hemp pressed cake (unfermented or fermented with *Rhizopus oligosporus*)	1 g + 5mL solvent (water or 80% ethanol), stirring (150rpm, 24 h, 27 °C)	• Spectro-photometric Analysis	• Depending on the solvent and fermentation duration: TPC (1.05–10.42 mg GAE/g DW) • Fermentation increased the bioavailability of some phenolics (optimal: 7 days)	• Antioxidant activity (DPPH, ABTS) • Antihypertensive activity: inhibition of angiotensin converting enzyme of 0.19–2.78 mg of captopril/g DW • Antidiabetic activity: 3.7%−92.4% inhibition of α-glucosidase	(Aktas et al. 2025)
Seeds from 2 locations (subtropical and temperate climate)	Defatting of 500 g with hexane Extraction by chloroform and methanol, or methanol only	• Spectro-photometric Analysis	• TPC (0.013–1.15 mg GAE/100 mg DW) and TFC (0.19–1.65 mg QE/100 mg DW) • Effect of environment (altitude and climate)	• Antioxidant activity: DPPH (26–80% inhibition), FRAP (0.48–250.81 µmol Fe (II) 100 mg^−1^ DW), Fe^2+^ chelation (22.37–31.5%) • DNA protective activity: all extracts prevent the DNA nicking	(Rashid et al. 2021)

*PE* petroleum ether, *RP-LC* reverse phase column liquid chromatography, *RP-MPLC* medium-pressure reverse phase column liquid chromatography, *CC* column chromatography, *HPLC* high performance liquid chromatography, *HSCCC* High-speed countercurrent chromatography, *NMR* nuclear magnetic resonance, *HR-MS* high-resolution mass spectrometry, *DAD* Diode array detector, *QTOF* Quadrupole-Time-of-Flight, *IR* infrared, *CT N-trans*-caffeoyltyramine, *CA* cannabisin A, *CB* cannabisin B, *TPC* total phenolic content, *GAE* gallic acid equivalent, *TAE* Tannic Acid Equivalents, *TFC* total flavonoid content, *QE* quercetin equivalent, *LUE* Luteolin equivalent, *CE* catechin equivalent, *RE* rutin equivalent, *TE* Trolox equivalent

**Table 3:** Studies using ultrasound assisted extraction of phenolic compounds from cannabis seeds and their by-products

Plant material (Variety/ region)	Extraction conditions	Analytical techniques (isolation/identification)	Main results		Reference
			**Phenolic content**	**Biological activity**	
Defatted kernels and hulls (2 varieties: Bama and Yunma No.1)	15 g + (1:10 w: v), 10 polar solvents (Water, Methanol, Ethanol, Acetone, and their aqueous mixtures) + Sonication (30min, manual stirring every 5 min)	• Spectro-photometric Analysis • Macroporous resin absorption • LH-20 gel chromatography • HPLC • HR-MS, NMR	• Hulls had higher TPC (0.92–13.93 mg GAE/100 g) than kernels (0.39–1.56 mg GAE/100 g) • Isolation of CT and CB using 60% ethanol from hulls	• Antioxidant activity: higher in hulls than kernels + IC_50_ of CT (9.42 µg/mL) and CB (11.17 µg/mL), • Cardioprotective activity: CT and CB inhibit human LDL oxidation	(Chen et al. 2012)
Defatted seeds	5 g + 25, 50, 75, 100 mL methanol: ace- tone: water (7:7:6), sonication (20, 30, 35 min), (40, 50, 60, 70 °C)	• Spectro-photometric Analysis	• TPC: 521.67–1542.03 mg GAE/100g FW • TFC: 8.39–25.27 mg LUE/100g FW	• Antioxidant activity: DPPH inhibition 30.92% and FRAP 16.7 µmol Fe (II)/g fresh weight using 50 mL solvent, 20 min, and 70 °C	(Teh and Birch 2014)
Seed meal (Helena, Serbia)	methanol/water (80:20 v/v), S/L ratio: 1:80 Sonication (RT, 10 min) + Maceration 2h	• Spectro-photometric Analysis • HPLC	• Hull Fractions (> 350 μm, > 250 μm): rich in CT (up to 287 mg/kg) and CB (up to 153 mg/kg) • Cotyledon Fractions (> 180 μm, < 180 μm): rich in catechin, and *p-*hydroxybenzoic acid	• Antioxidant activity: IC_50_-DPPH up to 5.29 mg/mL for coarse hull fraction (> 350 μm)	(Pojić et al. 2014)
Seeds, Oil, Flour (Fedora)	1 g + (10 mL + 6mL) of 80% methanol Vortex + sonication (dark, 4 °C, 30 min)	• Spectro-photometric Analysis • Reversed phase-HPLC–DAD	• TPC: 1540, 1078, and 23.5 mg CAE kg^−1^ for flour, seeds, and oil, respectively • Seed and flour: dominated by lignanamides • Oil: simple phenolics and cannabinoid derivatives	• Antioxidant activity (DPPH): 5.2, 4.7, and 0.36 Trolox milliequivalents for seeds, flour, and oil extracts (0.2 g mL^−1^), respectively	(Siano et al., 2018)
Seeds, oil, flour (Fedora)	10mL/g, 80% methanol Homogenization and vortex + sonication (30min) (repeated 3 times)	• Spectro-photometric Analysis • HPLC–DAD • HPLC–ESI–MS/MS	• TPC: 1709, 922, and 192 mg GAE kg^−1^ for flour, seeds and oil, respectively • Seeds and flour: high levels of phenylpropionamides and lignanamides • Oil: flavonoids, phenolic acids, and traces of cannabinoids	• Antioxidant activity: higher in seeds and flour (74 and 67% DPPH inhibition, respectively) • Cytotoxic activity: Oil had strong antiproliferative and apoptotic effects on colorectal cancer cells, but no significant cytotoxicity for seeds and flour	(Moccia et al. 2020)
Seeds	Defatting of 960 g with PE (9.6L, 2 h) Extraction with 70% ethanol (9.6L), sonication (3 times, 15 min)	• Liquid–liquid fractionation • Spectro-photometric Analysis • UPLC-QTOF-MS/MS	• TPC = 44.5–192.3 mg GAE/g extract depending on the fraction • Identification of 26 compounds in the ethyl acetate fraction (EtOAc)	• In vitro anti-tyrosinase activity: higher in the EtOAc fraction (IC_50_ = 24.5 µg/mL) • Cellular anti-tyrosinase activity: particularly higher for CT (IC_50_ = 0.8µM) with no toxicity of B16F10 melanoma cells	(Kim et al. 2021)
Whole and defatted seeds of 3 Italian hemp cultivars (Codimono, Carmaleonte and CS) during 2 years	8mL/g, Methanol acidified with 1 N HCl (80:20 v: v), sonication (30min)	• Spectro-photometric Analysis • HPLC	• TPC (3.8–5 mg FA/g) • CT (67.7- 108.9µg/g) and (208.8–723.4µg/g) in free and bound fractions, respectively • Some phenolic acids and flavonoids (*e.g*. caffeic, ferulic, syringic, and *p*-coumaric acids, naringenin, and epicatechin)	• Antioxidant activity (ABTS): 4.2–11.4 µmol Trolox equivalent (TE) g^−1^ (stronger effect of the year for whole seeds than defatted seeds)	(Menga et al. 2022)
Seeds	Defatting with hexane, S/L 1:5, extraction with ethanol, Sonication 4 times (30min each)	• Fractionation using silica gel CC • HPLC–UV-DAD • UHPLC-HR-MS/MS	• Determination of lignanamides-rich fraction (LnHS) • Phenylamides & Lignanamides are the most abundant (79%) • Flavonol glycosides constitute 2.8% (*e.g.* Quercetin pentoside, Kaempferol pentoside)	• Cytotoxic activity: Selective toxicity of LnHS against glioblastoma cells (DNA damage, inhibition of colony formation and migration). LnHS does not harm healthy fibroblasts	(Nigro et al. 2020)
Seeds of 7 industrial hemp varieties grown in Greece over three years	Defatting (10g) with hexane Cake + 80% methanol, sonication (30min, 65 °C)	• Spectro-photometric Analysis • HPLC–DAD	• TPC: 381.8—779.8 mg 100 g^−1^ • CT: 14.8—83.2 mg 100 g^−1^ • CA: 51.1- 159.1 mg 100 g^−1^	• Antioxidant activity: Up to 1066.3 and 806.8 mg TE 100 g^−1^ for ABTS and FRAP respectively for Futura in 2017. (Year had a more significant impact than Genotype)	(Irakli et al., 2019)
Seed cake	Preheating (140, 160, and 180 °C) for (5, 15, and 30 min), 9mL/g, 70% methanol, 80% acetone, and a mixture (MA) of 70% methanol 70% acetone at (1:1), Sonication (3times) for 1min	• Spectro-photometric Analysis • HPLC–DAD	• 80% acetone was the best solvent Depending on preheating temperature and extraction time: • CT: 0.61–0.91 mg CatE/g DW • CB: 0.88–1.20 mg CatE/g DW • Identification of Quercetin and some phenolic acids	Not studied	(Liang et al. 2018)
Seeds of Beldia and Critical from 4 regions (Morocco)	0.1g + 0.9 mL 90% methanol, vortex 1 min, sonication (30min), process repeated	• Spectro-photometric Analysis	• TPC: 134.57–199.90 mg GAE per 100 g seeds • TFC 39.4 to 69.54 mg QE per 100 g seeds • More pronounced effect of region than genotype	Not studied	(Taaifi et al. 2021)
Seeds of Beldia cultivar from Morocco	Defatting with PE 0.6g + 6mL (Acetone, methanol, water, and their mixtures), vortex 5 min and sonication 45min	• Spectro-photometric Analysis • HPLC–DAD/ESI-MS2	• Identification of 33 phenolic compounds • Depending on solvents: TPC: 6.86–50.19 mg GAE/g extract CT: 1.25- 33.83 mg/g extract CA: 2.41–21.65 mg CTE/g extract CB: 1.96–18.63 mg CTE/g extract • 50% acetone was optimal	• Antioxidant activity Using 50% acetone: 265.53, 36.25, 119.03, 69.46, and 68.91 mg TE g-1 extract for the TAC, DPPH, ABTS, FRAP, and CUPRAC tests respectively	(Benkirane et al., 2022)
Seeds of Beldia and Critical from 4 regions (Morocco)	Defatting with PE 0.6g + 6mL 50% acetone, vortex 5 min and sonication 45min	• HPLC–DAD/ESI-MS2	• Identification of 33 phenolic compounds • CT: 390.22–721.41 µg g^−1^ seeds • CA: 217.96–393.37 µg CTE g^−1^ • CB: 195.25–331.28 µg CTE g^−1^ • More pronounced effect of region than genotype	• Antioxidant activity IC_50_ = 1.83–4.14, 1.64–4.37, 2.45–6.02, 2.65–9.29 and 1.75–4.37 mg mL^−1^ of extract for the TAC, DPPH, ABTS, CUPRAC and FRAP tests, respectively	(Benkirane et al., 2023)
Seeds of ten industrial hemp cultivars (in Canada)	0.1g + (0.9 mL + 0.6 mL) 90%methanol, vortex, sonication (30min)	• Spectro-photometric Analysis	• TPC: from 1368 mg GAE/100 g (CRS-1) to 5160 mg/100 g (Anka). Significant effect of variety	Not studied	(Vonapartis et al. 2015)
4 Oil samples, 2 flour samples (milling &sieving of cake), 1 flour by-product (residue of the sieving process)	1 g + 1mL hexane, agitation, + 1 mL of MeOH/water (80:20 v/v), agitation 5 min, sonication 5 min, (Repeated twice)	• UHPLC-PDA/ESI–MS	• 50 phenolic compounds (9 phenolamides and 22 lignanamides) • Flour by-product the richest in: ✓ Total phenols: 1345.16 mg/kg ✓Hydroxycinnamic acid amides, lignanamides, and flavone glycosides: 450.67, 801, and 20.49 mg/kg, respectively ✓ CT: 270.24 mg/kg ✓ CA + CB: 159.65 mg/kg	Not studied	(Trovato et al. 2023)
Seeds from Dongbei, Guangxi, Inner Mongolia, and Shanxi in China	30 mg + 1mL chloroform: methanol (1:9, v/v), sonication (30min, 40 °C), Repeated	• Spectro-photometric Analysis	• TPC = 1.86–2.79 mg GAE/g, TFC = 3.45–5.88 mg RutinE/g • Effect of variety and region	• Antioxidant activity of hemp seed protein isolates (DPPH, ABTS, reducing power)	(Song et al. 2022)

*PE* petroleum ether, *HPLC/UHPLC* High/Ultra-high performance liquid chromatography, *DAD* Diode-array detector, *MS* mass spectrometry, *HR-MS* high resolution mass spectrometry, *ESI* electrospray ionization, *NMR* Nuclear magnetic resonance, *QTOF* Quadrupole-Time-of-Flight, *TPC* total phenolic content, *GAE* gallic acid equivalent, *CAE* caffeic acid equivalent, *TFC* total flavonoid content, *QE* Quercetin equivalent, *CatE* catechin equivalent, *TE* Trolox equivalent, *CT N-trans*-caffeoyltyramine, *CA* cannabisin A, *CB* cannabisin B

In addition to spectrophotometric assays, other studies employed advanced chromatographic and spectrometric techniques to accurately quantify individual phenolics and provide a more detailed and precise understanding of cannabis seed composition (thoroughly discussed in Section "Extraction, isolation, and identification of phenolic compounds from cannabis seeds" of this review). The predominant phenolamide in cannabis seeds is *N-trans*-caffeoyltyramine, while cannabisin A and cannabisin B are the most representative lignanamides. The extracted amounts of these three phenolic compounds, identified as signature molecules of cannabis seeds, are also reported in Tables 2 and 3, where available. The discrepancies in the reported phenolic content across studies could be attributed to several factors (thoroughly discussed in Sect. "Factors affecting phenolic compound content" of this review).

## Extraction, isolation, and identification of phenolic compounds from cannabis seeds

**Fig. 3:**
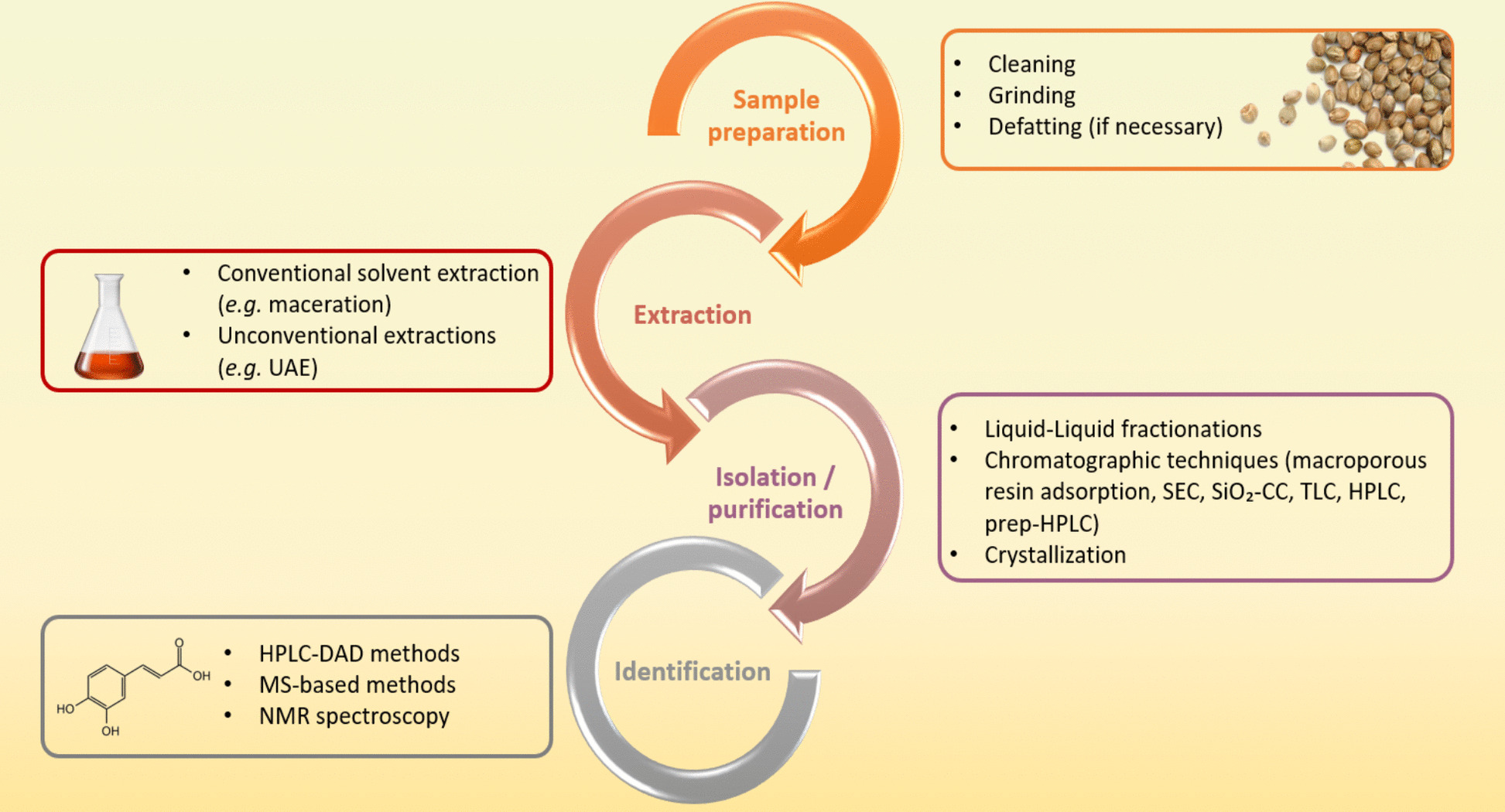
Flowchart highlighting the main methods employed during the analysis of phenolic compounds of cannabis seeds and by-products, from sample preparation to final characterization

The extraction of individual phenolic compounds from plant materials -including cannabis seeds- involves multiple steps. Figure 3 presents a structured flowchart summarizing the key stages and techniques used in the analysis of phenolic compounds from cannabis seeds and their by-products.

### Sample preparation

Before extraction, cannabis seeds must be properly prepared to ensure better extraction of phenolic compounds. First, the seeds should be cleaned to remove impurities or contaminations from other plant organs, such as stem or leaf fragments. This step is particularly important when seeds are collected directly from farmers or local markets. Next, the seeds must be properly crushed to increase the surface area, promoting solvent penetration into the plant matrix. The defatting step is also essential, as it removes the oil from the seeds, facilitating the subsequent extraction and quantification of phenolic compounds. Common solvents used in this step include hexane and petroleum ether (Benkirane et al. 2022; Menga et al. 2022; Rea Martinez et al. 2020; Yan et al. 2015), with some studies also using heptane (Lesma et al. 2014). In some cases, the defatting step could be skipped if the starting material is already defatted (cake/meal from the oil production industry), or contains minimal lipids (hulls).

### Extraction of phenolic compounds

The choice of extraction method affects both the yield and quality of the obtained phenolic compounds. Currently, a multitude of techniques are available, feasible and accessible, at laboratory, semi-industrial, or even industrial scales. These methods can be classified as conventional (*e.g.,* maceration) or unconventional (*e.g.,* ultrasound assisted extraction, microwave assisted extraction, supercritical fluid extraction) (Alara et al. 2021).

Most studies aiming to extract phenolic compounds from cannabis seeds (or their by-products), rely on conventional solvent extractions. The most commonly used solvents are methanol, ethanol, acetone, and their aqueous mixtures. In contrast, Mourtzinos et al. (2018) interestingly explored the use of a green solvent based on 2-hydroxypropyl-β-cyclodextrin and found encouraging results on the yields of phenolic compounds extracted from hemp flour after process optimization (Mourtzinos et al. 2018). The conventional solvent extraction often involves dynamic maceration where the plant material is soaked in a solvent for a specified period with agitation (stirring or shaking) to improve compound diffusion. Conventional extraction can also be performed using percolation (Yan et al. 2015) or under reflux (Zhou et al. 2018a, b). All these forms of traditional extraction generally take long durations, ranging from 1 to 24 h, with some studies extending up to 3 days (Yan et al. 2015) or even 10 days (Kim et al. 2023). In addition to being time-consuming, conventional extraction techniques present several notable disadvantages that limit their efficiency and sustainability. They often rely on toxic solvents, posing risks to human health and the environment, while generating hazardous waste. These methods are also costly consuming due to large solvent volumes and energy requirements. Moreover, they may exhibit limited selectivity, frequently co-extracting unwanted compounds. Collectively, these limitations have driven the search for greener, more efficient, and safer extraction alternatives. Several studies have used ultrasound-assisted extraction (UAE), with durations around 30 min in most cases (Chen et al. 2012; Irakli et al. 2019; Siano et al. 2018) and even reaching 5 min in the study of Trovato et al. (2023) and 1 min in that of Liang et al. (2018). However, all these UAE studies used an ultrasonic bath rather than an ultrasonic probe, although evidence from other plant matrices suggests that probes are more efficient (Jacotet-navarro et al. 2015). Tables 2 and 3 summarize the studies and key findings on the extraction of phenolic compounds from cannabis seeds (and their by-products) using conventional solvent extraction and ultrasound-assisted extraction, respectively.

Other unconventional extraction techniques, such as microwave-assisted extraction (MAE), supercritical fluid extraction (SFE), and enzyme-assisted extraction (EAE), have been widely explored to extract oil from cannabis seeds (Allay et al. 2024; Rezvankhah et al. 2019) or to extract some bioactive compounds from other cannabis organs, such as leaves and inflorescences (Drinić et al. 2018; Gallo-molina et al. 2019; Matešić et al. 2020). However, few studies were found applying these methods to explicitly investigate the extractability of phenolic compounds from seeds or their by-products. For example, Michailidis et al. (2021) applied SFE and UAE on hemp seed paste (obtained after oil cold-pressing) and successfully extracted phenolic compounds, including some phenolamides and lignanamides. Teh et al. (2014b) used MAE and Pulsed Electric Field (PEF) as pre-treatments for UAE to optimize the extraction of phenolic compounds from hemp seed cake powder. Yang et al. (2017) used a conventional method (Soxhlet) alongside three unconventional techniques (MAE, UAE, and SFE) to extract resin from cannabis seeds. They quantified 4 cannabinoids of interest but did not investigate other major classes of phenolic compounds. In another study, the authors performed ethanol maceration (48 h) and SFE (CO_2_, 40 °C, 400 bar, 60 min) from hulled seeds. The obtained extracts were compared in terms of antioxidant activity (DPPH, ABTS, gene expression of antioxidant enzymes) without quantifying phenolic compounds or even mentioning them in their discussion (Hong et al. 2015). Considering the multiple advantages of these innovative techniques over conventional solvent extraction, and the scarcity of studies on the cannabis seed phenolics, this topic seems to present a promising area for future research.

Beyond the extraction method used, it is noteworthy that some studies have followed typical protocols to improve the extraction of phenolic compounds from cannabis seeds and their by-products. For example, Chen et al. (2012) adopted a gradient elution extraction (0–100% ethanol) to selectively extract molecules based on their solubility. Sakakibara et al. (1995, 1992) used boiling water–ethanol (1:1) to promote extraction, while other authors used acid-alkaline hydrolysis to extract free and bound phenolic compounds and study them separately (Leonard et al. 2021a; Menga et al. 2022).

Overall, the extraction of phenolic compounds from cannabis seeds remains largely reliant on conventional solvent-based approaches, with limited adoption of innovative and green technologies. Ultrasound-assisted extraction represents the main investigated unconventional process and has demonstrated clear gains in efficiency, even though its implementation remains technically conservative. Other advanced methods have been scarcely applied to cannabis seeds and, when used, often without a clear focus on phenolic compounds. This collectively reveals a methodological gap and underscores the need for targeted development of selective, sustainable extraction processes tailored to cannabis seed phenolics.

### Isolation and purification of phenolic compounds

After extraction, the resulting extract usually contains a set of desired phenolic compounds along with other unwanted molecules. Separation and purification steps are then required to remove impurities and interfering compounds.

Liquid–liquid fractionations have been performed by several studies using solvents of increasing polarity to obtain separate fractions, containing apolar molecules, moderately polar molecules, and very polar molecules. The choice of the fraction recovered at the end of this step depends on the nature and polarity of the phenolic compounds sought. Sakakibara et al. (1995) followed the 50%-ethanol extraction by a fractionation using successively chloroform to eliminate lipophilic and non-polar compounds and *n*-butanol to extract polar phenolic compounds. Other studies used a fractionation in 3 successive steps with petroleum ether, ethyl acetate and *n*-butanol (after 75% ethanol extraction) (Wang et al. 2019; Yan et al. 2015). Kim et al. (2024) used fractionation with hexane, chloroform, ethyl acetate, and water. In another study, Rea Martinez et al. (2020) tested two distinct extraction and liquid–liquid fractionation processes and found that ethanol extraction (75%) followed by ethyl acetate and *n*-butanol fractionation was the most effective. In most studies, the ethyl acetate fraction was found to be the richest in phenolic compounds.

The studies that have successfully isolated phenolic compounds from cannabis seeds have used several different chromatographic techniques, ranging from the use of macroporous adsorbent resins to High Performance Liquid Chromatography (HPLC). Macroporous resins are high molecular weight polymers that selectively adsorb extract molecules and separate them based on their polarity and affinity to the resin. Due to its low cost, simplicity and efficiency, this method is typically used before progressing to other advanced chromatographic methods as in the works of Chen et al. (2012), Zhou et al. (2018a), and Sakakibara et al. (1995). Additionally, size exclusion chromatography also called gel permeation chromatography has been used by several researchers to further purify cannabis seed extracts. Sephadex LH-20 is a gel-like resin, very commonly used in this type of chromatography (Chen et al. 2012; Lesma et al. 2014; Sakakibara et al. 1991; Yan et al. 2015). It separates molecules according to their size and hydrophobic interactions. Silica gel column chromatography is a specific type of column chromatography where the stationary phase is silica gel. It is widely used to purify and separate compounds based on their polarity (Bourjot et al. 2016; Nigro et al. 2020). Thin layer chromatography is less used, but finds its place among the separation techniques employed by some researchers (Bourjot et al. 2016; Kim et al. 2023; Zhou et al. 2018b). In this technique, the stationary phase is a thin film of adsorbent material, usually silica, which is deposited on a plate (usually a glass or plastic plate).

High-performance liquid chromatography (HPLC), being the most efficient separation method, is often used by researchers as the final step in the purification and isolation of phenolic compounds. Preparative or semi-preparative HPLC are very practical for this purpose because the purified fractions can be collected and isolated for further analysis (Sakakibara et al. 1991; Wang et al. 2019; Yan et al. 2015). Preparative HPLC differs from analytical HPLC in terms of column diameter and mobile phase flow rate. The column in preparative HPLC is wider and the flow rate is higher, allowing a large volume of extract to be processed and a larger amount of purified product to be recovered. The final crystallization used in some studies further improves the purity of the isolated product, which is an asset for structural or biochemical studies. The process involves crystallizing the isolated compound under conditions that favor the formation of purified crystals (Wang et al. 2019).

The purification of compounds extracted from cannabis seeds or other plant matrices is generally carried out by combining several successive chromatographic techniques, while using several organic solvents depending on the polarity of the molecule to be purified. For instance, two phenolic compounds (*N-trans*-caffeoyltyramine and cannabisin B) were isolated from a 60% ethanolic extract of hemp seed hull using HPD-600 macroporous resin adsorption, Sephadex LH-20 gel chromatography, and high-performance liquid chromatography methods (Chen et al. 2012). Some cannabisins (A, B, C, D, E, F, G) were isolated by combining adsorption on Diaion HP-20 resin, silica gel chromatography, Sephadex LH-20 chromatography, preparative liquid chromatography (prepacked CIG Si-10 column), and preparative HPLC (Sakakibara et al. 1995). Similarly, Yan et al. (2015) developed a protocol combining reversed-phase column chromatography (RP-CC), Sephadex LH-20 gel chromatography, MCI gel chromatography and a final purification by HPLC to isolate several compounds, including cannabisins M, N, and O.

All these aforementioned methods, although laborious, offer a broad spectrum of extremely pure isolated compounds. However, other studies aimed to enrich a fraction in some phenolic compounds of interest instead of going as far as isolating a compound individually. In this context, after extraction with ethanol, Nigro et al. (2020) proceeded to fractionation by chromatography on a silica gel column with chloroform, ethyl acetate and methanol, to separate the compounds according to their polarity. The methanolic fraction was richer in lignanamides. Similarly, Zhou et al. (2018a) separated an ethanolic extract (70%) using a D101 macroporous adsorption resin column using H_2_O, followed by 75% and 95% ethanol successively to obtain the 75% ethanol fraction rich in phenylpropionamides (233.52 ± 2.50 μg/mg of extract) from hemp seeds.

### Identification of phenolic compounds

The identification of cannabis seed phenolic compounds relies on various analytical techniques, such as HPLC–PDA/DAD methods, mass spectrometry-based approaches, and Nuclear Magnetic Resonance (NMR) spectroscopy.

#### HPLC–PDA/DAD methods

High-performance liquid chromatography (HPLC) coupled with diode/photodiode detectors (DAD/PDA) has been widely used to separate and identify phenolic compounds in extracts from seeds (Babiker et al. 2021; Haddou et al. 2023; Irakli et al. 2019; Menga et al. 2022), hulls (Xu et al. 2024), or seed cakes (Liang et al. 2018). All these studies detected and quantified several phenolic compounds, mainly belonging to the class of phenolic acids and flavonoids because of the availability of their commercial standards. However, the major phenolic group of phenylpropionamides (HCAAs and lignanamides) were not identified, except for *N-trans*-caffeoyltyramine and cannabisin A and B. These three molecules were identified in some studies by HPLC–PDA/DAD due to the presence of standards (Liang et al. 2018; Menga et al. 2022; Xu et al. 2024) or based on their UV spectra, retention time and their remarkable peak size in the chromatogram (Irakli et al. 2019). Overall, HPLC–PDA/DAD methods provide valuable quantitative data but are limited in their ability to confirm structural details or detect compounds accurately.

#### MS-based techniques for phenolic compound identification

To detect a broader range of phenolic compounds (including hydroxycinnamic acid amides, lignanamides, and minor phenolics) with improved accuracy and sensitivity, while obtaining structural confirmation, many studies have used HPLC or UHPLC coupled with mass spectrometry (MS). Benkirane et al. (2022) applied HPLC–DAD/ESI-MS^2^ and successfully identified 33 phenolic compounds in cannabis seeds, with 5 HCAAs, 20 lignanamides, 4 phenolic acids, and traces of 4 cannabinoids. Similarly, Trovato et al. (2023) used UHPLC-PDA/ESI–MS to compare oils, flours, and flour by-products, identifying 50 phenolic compounds, including 31 phenylpropionamides (9 HCAAs and 22 lignanamides), 4 phenolic acids, 1 lignan, 2 flavones, and 12 cannabinoids.

Advanced MS techniques, namely high-resolution mass spectroscopy (HR-MS), enable the measurement of the exact mass of ions with high accuracy, which is essential for compound identification and structural elucidation. HR-MS spectrometers use mass analyzers such as Time-of-Flight (TOF), Quadrupole-Time-of-Flight (QTOF), and Orbitrap. In this context, Nigro et al. (2020) used UHPLC-ESI-QTOF-MS/MS for the detailed characterization of cannabis seed extracts and detected 7 Phenylamides, 25 Lignanamides, and 7 Flavonol Glycosides. Using HR-MS/MS and UV-DAD, these authors confirmed the structural diversity of lignanamides, highlighting their complex lignan core (arylnaphthalene, benzodioxane, β-aryl ether, etc.), while discussing in depth their fragmentation patterns. Similarly, Leonard et al. (2021a) used HPLC-ESI-QTOF-MS/MS to detect 26 different phenylpropionamides (HCAAs and lignanamides). Other researchers have used UHPLC with orbitrap spectrometers, such as Rea Martinez et al. (2020), who identified 11 phenolic acids, 8 flavonoids, 5 HCCAs, 3 lignanamides, and traces of 3 cannabinoids.

Surprisingly, some studies have not be able to identify a large number of phenolic compounds despite using advanced identification techniques. For example, Frazzini et al. (2024) identified a relatively low number of individual phenolic compounds (phenolic acids and flavonoids only) in dehulled hemp seeds, although the method used was very sensitive and accurate (HPLC-Q-TOF–MS/MS). This may be attributed to the dehulling process, as dehulled seeds may naturally contain fewer detectable polyphenols, or to the extraction method used, where the QuEChERS step could lead to the loss of some hydrophilic polyphenols. Similarly, Aloo et al. (2023) used UHPLC-Q-TOF–MS/MS to accurately identify and quantify bioactive compounds from hemp seeds and stems. However, they only identified and quantified phenolic acids and flavonoids with no detection of phenylpropionamides (HCAAs and lignanamides). The study by Alonso-Esteban et al. (2022) used the Dionex Ultimate 3000 UHPLC system coupled with a DAD and ESI–MS to identify phenolic compounds from the seed. Although this method is standard and provides valuable information, only ferulic acid-hexoside and syringic acid were identified, potentially missing other important phenolic compounds.

It is important to highlight that high-resolution mass spectroscopy was also used to conduct untargeted metabolomic studies. They are of great importance in the search for new compounds and provide a broader overview of the phytochemical diversity of cannabis seeds. Frassinetti et al. (2018) used HR-MS-based metabolomics to profile major metabolites, including polyphenolic amides (caffeoyltyramine, cannabisin A, B, and C), fatty acids, sugars, amino acids, and cannabinoids. Ning et al. (2022) performed UHPLC-QQQ-MS/MS metabolomic profiling, identifying 1,001 metabolites, including 201 flavonoids, 86 alkaloids, and 149 phenolic acids. In a recent such study conducted on 52 germplasm accessions, researchers discovered an entirely new molecular family in cannabis seeds (cinnamic acid glycosyl sulphates) (Padilla-gonzález et al. 2023). These studies highlight the importance of metabolomics to capture compounds often overlooked by targeted HPLC–MS approaches.

#### Nuclear magnetic resonance spectroscopy

In addition to mass spectroscopy, nuclear magnetic resonance (NMR) spectroscopy has played a crucial role in confirming the structures of phenolic compounds. Chen et al. (2012) used HR-MS spectra, NMR spectra, and UV data to validate the presence of *N-trans*-caffeoyltyramine and cannabisin B. Kim et al. (2024) further characterized several lignanamides using ^1^H- and ^13^C NMR with mass spectrometry. Yan et al. (2015) used NMR, HR-MS, UV, and InfraRed spectroscopy to determine the structure of novel lignanamides, such as cannabisins M, N, and O.

Overall, the presence of unidentified peaks in several studies suggests that cannabis seed extracts contain additional bioactive compounds that are not yet documented. Future studies would be necessary, while integrating multiple isolation-identification techniques for a more comprehensive characterization.

## Factors affecting phenolic compound content

### Effect of extraction method, extraction conditions, and choice of solvent

The extraction method, along with the selected conditions and solvent, plays a crucial role in determining the content of phenolic compounds extracted from a plant matrix, including cannabis seeds. Teh and Birch (2014) found that ultrasound-assisted extraction gave the best results (TPC, DPPH, FRAP) compared to conventional solvent extraction, under the same experimental conditions (Teh and Birch 2014). These authors determined that the solvent volume was a major influencing factor in UAE, followed by extraction temperature and time (Teh and Birch 2014). Michailidis et al. (2021) compared Supercritical Fluid Extraction (SFE) and Ultrasound Assisted Extraction (UAE) and found that CO_2_ SFE using 20% ethanol as co-solvent led to the richest extract in phenolic metabolites from hemp seed paste.

The extractability of phenolic compounds in a given solvent depends mainly on its polarity. Aware of this, several studies have tested a wide range of solvents to determine which one favors the extraction of phenolic compounds from cannabis seeds, their cake or their hull. Teh et al. (2014a, b) tested several solvents and found that total phenolic (TPC) and total flavonoid (TFC) contents ranged from 167 to 733 mg GAE/100 g and from 0.23 to 27 mg LUE/100 g fresh weight, depending on the solvent, with methanol-acetone–water (6:6:7 v:v:v) being optimal. In the same context, hexane, dichloromethane, ethanol and water were used to extract phenolic compounds from cannabis seeds; the ethanolic extract was the richest, achieving 130 µg GAE/mg extract (Haddou et al. 2023). Chen et al. (2012) compared the effect of 10 polar solvents and chose 75% acetone as optimal. Similarly, another comparative study conducted on hemp seed cake found that 80% acetone extracts had the highest total phenolic content (TPC), followed by 70% acetone:70% methanol (1:1) and finally 70% methanol (Liang et al. 2018). These authors also demonstrated that preheating temperature and exposure time improved the TPC for all solvents (Liang et al. 2018).

Interestingly, Benkirane et al. (2022) used a simplex lattice mixture design to select the ideal solvent for the extraction of phenolic compounds from cannabis seeds. This approach is a variant of Response Surface Methodology (RSM), a powerful statistical tool used to model and optimize a response based on multiple experimental variables. The authors found that a 1:1 acetone to water ratio was optimal, yielding a total phenolic content of 53.65 mg GAE per g of extract, with superior antioxidant activities in several assays (TAC, DPPH, ABTS, FRAP, and CUPRAC) (Benkirane et al. 2022). The same approach, using this time the Simplex centroid mixture design, was also tested by Aazza on *Cannabis sativa* plant residues, and proved the performance of ethanol as a solvent (Aazza 2021). In another study, RSM was used to optimize the experimental conditions of extraction of phenolic compounds from hemp flour using a green solvent (2-hydroxypropyl-β-cyclodextrin (CD)). The extraction under optimized conditions (32.1% CD concentration (w/v), solid/solvent ratio 1/15.2 g/mL, and 28 °C extraction temperature) gave the highest TPC and the strongest antioxidant activity than conventional solvents, such as methanol, ethanol, and water (Mourtzinos et al. 2018). Future research should focus on the use of these statistical approaches rather than full factorial experiments. Their application could help identify the best conditions for extracting bioactive compounds (maximizing yield and extract quality), while minimizing the number of tests and reducing experimental costs. Furthermore, they promote more sustainable and efficient extractions by considering the complex interactions between variables, which will potentially revolutionize extraction processes across various research fields.

### Effect of genotype, environment, and their interaction

The phenolic content may vary depending on genetic and/or environmental factors (climate, altitude, cultivation practices, etc.). Vonapartis et al. (2015) conducted an experimental study in Canada targeting the composition of ten industrial hemp cultivars and demonstrated a significant effect of the variety on TPC with contents ranging from 1368 to 5160 mg GAE/100 g for the CRS-1 and Anka varieties, respectively. Menga et al. (2022) demonstrated the effect of genotype, cultivation year, and their interaction on several nutritional and antioxidant properties of whole and defatted cannabis seeds of two Italian varieties. The effect of the environment in terms of altitude and climate was also studied on two cannabis accessions. High-altitude, temperate climate accession seeds have an advantage over low-altitude, subtropical climate accession seeds, in terms of nutraceutical importance, phenolic compounds (TPC, TFC), and antioxidant activity (DPPH, FRAP, metal chelation) (Rashid et al. 2021). In another study, researchers found that agronomic practices, such as planting density and pre-sowing fertilization massively affected the relative content of each class of phenolic compounds in the extracted oil. Hemp seeds from soils without pre-sowing fertilization and with a crop density of 60 plants/m^2^ had the highest production of polyphenols, while their abundance could be compromised when the plant density was reduced by half (Faugno et al. 2019). These agronomic practices influencing phenolic accumulation in oil are likely to have a similar impact on seeds.

Taaifi et al. (2021) analyzed two cannabis varieties (Beldia and Critical) grown in 4 different regions of northern Morocco to study the effect of variety, growing area, and variety × growing area interaction on several parameters. The authors found that the nutritional composition is rather affected by the genotype, while the TPC is more dependent on the production site. Seeds produced in the Jebha region are the richest in phenolics for both varieties (198.88 −195.18 mg GAE 100 g^−1^), while the lowest values ​​were observed in the Ratba region (135.90 and 177.63 mg GAE 100 g^−1^) (Taaifi et al. 2021). The same research team conducted another more in-depth study involving the same plant material and growing regions to study their phenolic profile using HPLC–DAD/ESI-MS^2^. The obtained results confirmed that the content of phenolic compounds was mainly affected by the geographical location and its interaction with the genotype factor (Benkirane et al. 2023). Irakli et al. (2019) also investigated the effect of genotype, growing year, and their interaction on the phytochemical composition of cannabis seeds of 7 varieties grown in Greece for 3 consecutive years. They found that the year had a more significant impact on phenolic compounds and antioxidant activity than genotype. However, protein, oil, and fatty acid composition were mainly affected by genetic factors.

### Effect of seed treatment/processing

The processing and treatment of cannabis seeds can significantly impact their phenolic compound profile, influencing both composition and concentration. Various techniques will be discussed in this section, such as defatting, dehulling, roasting, extrusion, and fermentation.

#### Defatting and grinding

Defatting is the process of removing fats or lipids from plant materials. The high fat content in hemp seeds could interfere with the extraction and analysis of polyphenols. To minimize this issue and ensure accurate results, several studies remove the oil content before extracting phenolic compounds from whole seeds or kernels (Benkirane et al. 2022; Chen et al. 2012; Frazzini et al. 2024; Lesma et al. 2014; Nigro et al. 2020).

Seed grinding is a mechanical process that reduces seeds into smaller particles, forming a powder. It enhances the extraction of bioactive compounds by increasing the surface area available for extraction. A recent study investigated a novel grinding technique (jet milling) and studied the effect of particle size on ​​the dissolution of key phenolic compounds from hemp seed hull (*N-trans*-caffeoyltyramine, Cannabisin A, and Cannabisin B). Jet milling significantly reduced the particle size from coarse powder (HSSP1) to superfine powder (HSSSP). It improved the physicochemical properties of the resulting powder (higher specific surface area, better dispersion, and wettability), which facilitated the penetration of solvents, resulting in higher dissolution of polyphenols and better antioxidant activity. Based on FTIR analysis, jet milling changed the molecular structure of hemp seed hull powder by breaking hydrogen bonds, exposing more functional groups, and increasing amorphization (Xu et al. 2024).

#### Dehulling

Seed dehulling (also called hulling) is a process of separating and removing the outer shell (or hull) of the seed in order to access the inner kernel or endosperm. Hulling produces hemp hearts (or kernels), which are softer and easier to consume, but the absence of the hull reduces the fiber and phenolic content. Indeed, phenolic compounds are asymmetrically distributed in the cannabis seed, with hulls being the richest (Alonso-Esteban et al. 2022; Chen et al. 2012). This distribution of phenolic compounds is probably related to their role in defense against environmental stresses, given that hulls are the first line of defense against external aggressions, while kernels are more optimized for the storage of nutrients for germination.

Chen et al. (2012) simultaneously analyzed the hulls and kernels of seeds from two cannabis varieties (Bama and Yumna). The hulls exhibited higher TPC and antioxidant activity compared to kernels, which were rather rich in oil (50%) and proteins (67%). For this reason, the authors used the hulls to successfully isolate two major compounds, *N-trans*-caffeoyltyramine and cannabisin B (Chen et al. 2012). Alonso-Esteban et al. (2022) also confirmed these compositional differences. They showed that whole hemp seeds are significantly richer in fiber than hulled seeds, which have a higher fat and protein content. Their analysis included whole seeds of eight known hemp varieties and eight hulled seed samples. However, as the hulled seeds examined were commercial samples with unknown varietal information, the potential influence of genetic factors on seed composition could not be assessed. A comparison using identified varieties for both whole and hulled seeds would provide a better understanding of the specific impact of hulling on seed composition.

In another study, the cake from mechanical pressing of hemp seeds (Helena variety) was milled to obtain flour, subsequently fractionated by sieving (Pojić et al. 2014). Two groups of fractions were identified: coarse fractions with particle sizes > 350 and 250–350 μm, containing hull particles, and fine fractions with particle sizes 180–250 and < 180 μm, containing mainly cotyledon (kernel) particles. Cotyledon-rich fractions were significantly richer in proteins (41–44%), lipids (15–18%) and sugars (3–5%) compared to hull-rich fractions which had higher crude fiber content (21–29%). In addition, hull fractions were rich in cannabisin B and *N-trans*-caffeoyltyramine. However, cotyledon fractions were rather rich in catechin and *p*-hydroxybenzoic acid. This distribution of phenolic compounds suggests that different fractions could be selectively used to extract specific bioactive compounds for nutraceutical or pharmaceutical applications. This study therefore introduces sieving as an innovative fractionation approach. Indeed, it is a simple and cost-effective method to concentrate valuable nutrients and bioactive compounds. It enhances potential industrial applications by targeting specific fractions for different uses (Pojić et al. 2014).

#### Germination/sprouting

Germination/sprouting is a practice known for its ability to improve the nutrient profile of seeds as well as their phenolic profile. During germination, the seed transitions from dormancy to metabolic activation, leading to increased production of several phytochemicals necessary for its “early” development, including polyphenols.

Cannabis shoots aged 3 and 5 days have a higher content of polyphenols, flavonoids and flavonols than seeds (Frassinetti et al. 2018). The content of total polyphenols increases from 2.33 mg GAE/g DW in seeds to 5.04 and 6.16 mg GAE/g DW in 3- and 5-days old shoots. Similarly, flavonoids increase from 2.93 to 4.40 and then 5.32 mg QE/g DW, and flavonols from 0.85 to 2.28 and then 2.45 mg QE/g DW. The same observation was detected in the study of Aljuhaimi et al. (2024). Cannabis seeds germinated at 3 days presented higher values ​​than the control (non-germinated) in terms of total polyphenols (29.97 vs. 47.47 mg GAE/100g DW), flavonoids (28.76 vs. 44.49 mg QE/100g DW) and antioxidant capacity (1.39 vs. 2.30 mmol TE/kg DW). Recently, Pitiviroj et al. (2024) investigated the effect of germination on cannabis seeds by analyzing the general metabolome, while focusing on two unique flavonoids of *Cannabis sativa* (cannflavin A and B), which are absent in seeds. They detected that the TPC increased significantly during germination, from 44.40 mg/kg (day 0) to 93.94 mg/kg (day 3). In addition, the concentrations of cannflavin A and B increased progressively, reaching a peak at 72 h. All these data highlight the enrichment of cannabis seeds in bioactive compounds during germination, which is useful for the development of functional foods.

#### Extrusion

Extrusion is a mechanical process commonly used in the food industry. It consists of passing a material, such as seeds, through a screw under the effect of heat, pressure and shear. This process was tested on the cake and hulls of cannabis seeds of the CRS1 variety. The total phenolic content in the cake ranged from 0.385 to 0.906 mg GAE/g sample, depending on extrusion parameters. Lower moisture (30%) and higher screw speed (300 rpm) significantly increased the proportion of free polyphenols (Leonard et al. 2021a). Extrusion can therefore improve the bioavailability of certain phenolic compounds by breaking the bonds with insoluble matrices. Similarly, extrusion of hulls was responsible for increasing the total phenylpropionamide content from 25 to 78% (Leonard et al. 2021b).

#### Heat-involving processes

Heat treatment is considered among the main factors that can alter the level of phytochemicals in foods before consumption. Some studies have investigated the effect of some processes involving temperature, such as roasting and boiling, on the composition of phenolic compounds of cannabis seeds. Babiker et al. (2021) found that roasting can significantly increase the content of proteins, oil, some minerals, total phenolics, total flavonoids, and DPPH radical scavenging. Phenolic profile analysis by HPLC showed the effect of roasting time on the individual content of some detected phenolic acids and flavonoids. A roasting time of 14 min at 160 °C could be considered the most suitable duration. In addition, Mansouri et al. (2023) studied by RSM the effect of roasting seeds on the quality of the resulting oil after cold extraction. They found that roasting at 163 °C for 15 min was responsible for an increase in total phenols of the oil from 64 to 121 mg GAE/Kg and an improvement in antioxidant activity from 43% to 72% DPPH inhibition. In addition, roasted hemp seeds showed the appearance of 21 new aromatic compounds sought for their positive attributes, such as pyrazines and aldehydes, responsible for roasted, nutty and almond odors. Boiling of cannabis seeds also affected total phenol, flavonoid and antioxidant capacity values ​​(Aljuhaimi et al. 2024). Seeds boiled at 100 °C for 20 min demonstrated superiority (compared to the control) in terms of TPC (29.97 vs. 51.52 mg GAE/100 g DW), TFC (28.76 vs. 65.05 mg QE/100 g DW) and antioxidant activity (1.39 vs. 2.46 mmol TE/kg). Phenolic profile analysis showed the improvement of the content of some compounds, such as catechin (11.42 vs 44.16 mg/100 g), rutin (7.27 vs 16.18 mg/100 g), and quercetin (3.4 vs 8.88 mg/100 g). This improvement with respect to heat treatment may be due to the thermal disruption of membranes and cell walls, which makes phenolic compounds more accessible to extraction. Temperature may also promote the hydrolysis of some bonds that bind phenolic compounds to other molecules, thus improving their solubility.

#### Fermentation

Fermentation is a widely used process in the food industry to bio-transform foods and increase their nutritional quality and improve their digestibility. Aktas studied the solid-state fermentation of cannabis seed cake using *Rhizopus oligosporus*. This process significantly increased TPC, antioxidant activity, and essential amino acids, with values ​​varying depending on extract type and fermentation duration. Fermentation improved the bioavailability of some compounds, reaching an optimum at 7 days (Aktas et al. 2025). Some bioconversions performed by this enzyme during fermentation, such as methylation, carboxylation, sulfate conjugation, hydroxylation, and oxidation, may be the origin of new antioxidant compounds, thus contributing to the improvement of antioxidant activity.

Overall, the extractability and final content of phenolic compounds from cannabis seeds result from a complex interplay between extraction-related parameters, biological variability, and seed processing conditions. Method selection, solvent polarity, and operating conditions strongly modulate phenolic recovery, while statistical optimization tools increasingly demonstrate their value in capturing interactions between variables and improving efficiency. At the same time, genotype, environment, and their interaction introduce substantial intrinsic variability in phenolic accumulation, often exceeding methodological effects. Finally, seed processing steps (ranging from defatting and dehulling to germination, extrusion, thermal treatment, and fermentation) can either concentrate, transform, or enhance phenolic bioaccessibility by modifying tissue structure and phenolic binding forms. Together, these factors explain the wide variability reported in the literature and emphasize the need for integrated approaches that jointly consider biological origin, processing strategy, and extraction design when valorizing cannabis seed phenolics.

## Biological activities of cannabis seeds and potential applications

In this section, the different biological activities of cannabis seeds and their phenolic compounds are presented and discussed, as well as their potential applications. Key findings are summarized in Tables 2 and 3. Most studies investigating these biological activities report effects using either total extracts or isolated compounds. While total phenolic content (TPC) can provide a general estimate of phenolic abundance of an extract, it does not identify which specific molecules are responsible for observed activities. Moreover, the commonly used Folin-Ciocalteu assay primarily reflects reducing capacity rather than true phenolic specificity, which can lead to overestimation or misinterpretation of phenolic content. Therefore, correlations between TPC and bioactivity should be interpreted with caution, as extract-level effects may reflect additive or synergistic interactions among multiple compounds. Where available, we highlight results from isolated phenolics to clarify compound-specific activity. In the next subsections, the biological activities of cannabis seeds are presented separately for clarity, although they largely arise from interconnected or overlapping mechanisms.

### Antioxidant activity

Most studies are primarily interested in evaluating the antioxidant activity of plant extracts given its importance and its direct impact on human health. Indeed, oxidative stress is involved in several biological processes and chronic diseases, such as cardiovascular diseases (atherosclerosis), neurodegenerative diseases (Alzheimer's, Parkinson's), cancers (DNA mutations), and some metabolic syndromes (insulin resistance and complications of diabetes) (Lourenço et al. 2019).

A multitude of tests are available to evaluate the antioxidant activity of plant extracts. Chemical in-vitro tests are the most used and are based on the evaluation of free radical scavenging power (DPPH, ABTS, ORAC), reducing power (FRAP, CUPRAC), metal chelating power (Fe^2+^ or Cu^2+^), or lipid peroxidation (TBARS). Biological in-vitro tests are also frequently used such as the assay of gene expression of some antioxidant enzymes. In addition, the antioxidant activity of plant extracts can be evaluated by other more complex and physiologically relevant approaches, namely ex-vivo or in-vivo tests.

Cannabis seed extracts exhibit strong antioxidant properties in both in-vitro and ex-vivo models. Frassinetti et al. (2018) reported in-vitro antioxidant activity of hemp seeds, with approximately 40% DPPH radical scavenging and an ORAC value of 127 μmol TE/g fresh weight. In addition, the cellular antioxidant activity assay (CAA-RBC) and the hemolysis assay, both performed on an ex-vivo human erythrocyte system, demonstrated the ability of seeds to protect cells from oxidative damage (CAA = 82; 52% hemolysis inhibition) (Frassinetti et al. 2018). Hong et al. (2015) found that cannabis seed extracts (ethanolic and supercritical) showed significant radical scavenging activity in in-vitro assays (IC_50_ of 66.6–99.9 µg/mL and 9.2–10.7 mg/mL, respectively for ABTS and DPPH). To explore the cellular effects, the study examined the gene expression levels of antioxidant enzymes, including superoxide dismutase (SOD), glutathione peroxidase (GPx), and catalase (CAT), in HepG2 cells exposed to hydrogen peroxide (H₂O₂)-induced oxidative stress. Treatment with seed extracts enhanced the expression of SOD, GPx, and CAT genes, compared with the control (Hong et al. 2015). In another study, the superfine powder of cannabis seed hulls showed high DPPH and ABTS radical scavenging activity, with IC_50_ values ​​of 36.13 μg/mL and 32.30 μg/mL, respectively. Moreover, pretreatment with this extract at 100 µg/mL significantly reduced reactive oxygen species (ROS) production (from 3.97 to 1.95 compared to control), lipid peroxidation (malondialdehyde levels from 9.83 to 6.28 nmol/mg protein), and lactate dehydrogenase (LDH) leakage index (from 32.89 to 19.76%), while increasing the activity of the antioxidant enzyme SOD (from 13.14 to 16.24 U/mg protein) in H_2_O_2_-induced oxidative stress in HUVEC cells (Xu et al. 2024). All these studies suggest that cannabis seed extracts may potentially offer protective effects against oxidative stress-related cell damage.

The improvement of the antioxidant activity of an extract has always been accompanied by the improvement of the extractability of phenolic compounds following the optimization of the extraction conditions or solvent, or thanks to specific treatments, such as extrusion and roasting (Babiker et al. 2021; Benkirane et al. 2022; Leonard et al. 2021a; Teh et al. 2014a). Indeed, correlation analyses always confirm that the content of phenolic compounds is the main factor contributing to the antioxidant activity of cannabis seeds (Benkirane et al. 2022; Chen et al. 2012; Hwang et al. 2025; Irakli et al. 2019). This relationship was further supported by Ning et al. (2022) using weighted gene co-expression network analysis, in which 1001 metabolites were identified and clustered into six color-coded co-expression modules. Among these, three modules (brown, yellow, and red) were most strongly associated with antioxidant activity. Notably, specific phenolic acids (*e.g.,* feruloyl syringic acid) and alkaloid-like phenolics (*e.g., N-trans*-feruloyl-3′-O-methyldopamine) were commonly represented across all three modules. Additionally, the brown and yellow modules were particularly enriched in flavonoids, including chrysoeriol, kaempferol, and luteolin derivatives in the brown module, and quercetin and isorhamnetin derivatives in the yellow one. These finding reinforce the central contribution of phenolic compounds to the antioxidant potential of cannabis seeds.

This close relationship between phenolic compounds and antioxidant activity is proven by several studies that tested the antioxidant activity of some individual phenolic compounds after their isolation from the entire extract. *N-trans*-caffeoyltyramine and cannabisin B showed good DPPH free radical scavenging ability (IC_50_ = 9.42 µg/mL and 11.17 µg/mL respectively) and good ability to inhibit human LDL oxidation, proving their cardioprotective effect (Chen et al. 2012). Cannabisin F at 10 and 15 µM significantly reduced the production of cellular reactive oxygen species (ROS) by activating the Nrf2/HO-1 signaling pathway in LPS-stimulated BV2 microglial cells (Wang et al. 2019). These three molecules (caffeoyltyramine, cannabisin B, and cannabisin F) also showed significant antioxidant activity assessed by the ORAC test (8.9–9.8 µmol TE/µmol), followed by cannabisin I and M (4.3–5 µmol TE/µmol), indicating their potential in combating oxidative stress (Bourjot et al. 2016). In the same context, Yan et al. (2015) after isolating and evaluating 14 phenolic compounds from cannabis seeds, detected eight molecules with potential antioxidant effect assessed by DPPH, ABTS, and ORAC, with cannabisin D having an activity comparable to that of quercetin (IC_50_-DPPH 23.9 *Vs.* 25.5 µM, respectively) (Yan et al. 2015). Overall, all these studies demonstrate the antioxidant activity of phenolic compounds from cannabis seeds and highlight their potential in combating oxidative stress and supporting human health.

### Anticancer activity

Anticancer activity refers to the ability of an extract or compound to inhibit the growth, induce apoptosis, or prevent the spread of cancer cells. Several studies have tested cannabis seeds for their anticancer potential, revealing effects that are often cell-type dependent, extract-specific, and mechanistically diverse.

Frassinetti et al. (2018) analyzed the antimutagenic potency of cannabis seeds against H_2_O_2_-induced mutagenesis in an ex-vivo model of *Saccharomyces cerevisiae* yeast cells. At a non-cytotoxic concentration of 1 mg/mL, seed extracts significantly reduced DNA mutations and oxidative damage in cells, as evidenced by decreased mitotic gene conversion and point reverse mutation rates. These finding suggest a potential protective genotoxic effect, useful against certain cancers or diseases related to DNA mutations, although further in-vivo validation is needed. In the same context, Moccia et al. (2020) found that cannabis seed oil extracts (100 µg/mL) exhibited significant antiproliferative effect on colorectal cancer cells (Caco-2). This effect was associated with the induction of apoptotic cell death mediated by caspase-3 activation (approximately twofold compared to untreated cells), despite the lower phenolic content of the extract. However, cannabis seeds and cake had no significant cytotoxicity against this cancer line. Broader screenings across multiple cannabis varieties demonstrated that anticancer effects are dependent on the variety, presence of the hull, and cancer cell line. Alonso-Esteban et al. (2022) investigated extracts of whole and hulled cannabis seeds from several varieties regarding their cytotoxic effect on several cancer cell lines, namely MCF-7 (breast adenocarcinoma), NCI-H460 (non-small cell lung cancer), HeLa (cervical carcinoma), and HepG2 (hepatocellular carcinoma). The cytotoxic activity was particularly marked in the whole cannabis seed of the Bialobrzeskie variety against NCI-H460 non-small cell lung cancer (half-maximal growth inhibition concentration GI_50_ = 47 μg/mL). However, hulled seeds of the same variety showed no activity against any of the tested cancer cell (GI_50_ > 400 μg/mL). Most extracts tested did not significantly affect the viability of PLP2 non-tumor primary liver cells, which is a good indicator of their safety (Alonso-Esteban et al. 2022). A lignanamide-enriched fraction from cannabis seeds was tested by Nigro et al. (2020) on U-87 glioblastoma cells (brain cancer) and non-tumorigenic human fibroblasts. It significantly inhibited cancer cell proliferation, increasing DNA damage, and inhibiting colony formation and migration, without affecting normal fibroblasts. This selective toxicity suggests a possible role in cancer treatment and chemotherapy. Mechanistically, the fraction acted by blocking the autophagy while activating the apoptotic pathway via down-regulation of ULK-1 and Beclin-1, inhibition of Bcl-2, and reduction of AKT phosphorylation (Nigro et al. 2020). At the compound level, isolated lignanamides also show anticancer activity. Chen et al. (2013) demonstrated that cannabisin B induces autophagic cell death in HepG2 hepatocarcinoma cells, through AKT/mTOR pathway inhibition and S-phase cell cycle arrest. Molecular docking studies suggest that other lignanamides, including cannabinins M and N, could be potential therapeutic candidates against P-glycoprotein, a key efflux transporter implicated in drug resistance in cancer cells (Kazemi et al. 2021).

Overall, cannabis seed extracts and their phenolic compounds, including lignanamides, exhibit promising anticancer potential through multiple mechanisms, such as apoptosis induction, and autophagy modulation. However, these effects are highly variable and context-dependent, influenced by extract type, compound composition, and cancer cell line. Differences between whole extracts and isolated compounds further emphasize the need for standardized extraction methods, mechanistic studies, and reproducible in-vivo evaluations to fully validate their therapeutic potential.

### Neuroprotective activity

Zhou et al. (2018a) used a mouse model where neuroinflammation and cognitive deficits were induced using lipopolysaccharide (LPS) after two weeks of administration of a phenylpropionamide-rich extract to these mice (1 and 2 g/kg/day). Results showed notable improvements in learning and spatial memory based on Morris Water Maze Test. In addition, there was a significant reduction in pro-inflammatory cytokines (IL-1β, IL-6, and TNF-α) in brain tissue (at low dose 1 g/kg only), and an attenuation in LPS-induced neuronal damage in the hippocampus (Zhou et al. 2018a). In a transgenic mouse model of Alzheimer’s disease (APP/PS1), phenylpropionamides improved memory, reduced amyloid-β (Aβ) accumulation, and decreased oxidative stress, suggesting their potential for mitigating neurodegeneration (Yang et al. 2024). In a Parkinson’s disease model (MPTP-induced mice), phenylpropionamides protected dopaminergic neurons in the substantia nigra, improved motor function, and activated autophagy to reduce α-synuclein aggregation, a key hallmark of this disease (Jiang et al. 2024). Collectively, these three studies highlight the potential of phenylpropionamides as natural therapeutic agents for neurodegenerative diseases, offering cognitive protection, anti-inflammatory benefits, and neuronal survival support through distinct molecular pathways. In another study, Leonard et al. (2021a) found that extruded cannabis seed cake increased the inhibition of acetylcholinesterase (AChE), an enzyme that stops nerve signal transmission at cholinergic synapses. This AChE inhibition activity appears to be further influenced by cannabisins A-C, M according to correlation analyses (r > 0.8, *p* < 0.01).

The independent effect of phenolic compounds purified from cannabis seeds has been extensively studied. *N-trans*-caffeoyltyramine, 3,3′-demethyl-heliotropamide, and 3,3′-demethyl-grossamide significantly inhibited acetylcholinesterase in-vitro (with inhibition percentages of 83.28, 62.84, and 87.37%, respectively) (Yan et al. 2015). Cannabisin F and grossamide exert neuroprotective effects by significantly suppressing the production of pro-inflammatory cytokines (IL-6, TNF-α) in lipopolysaccharide-stimulated BV2 microglial cells (Luo et al. 2017; Wang et al. 2019). This effect is mediated by inhibition of the NF-κB signaling pathway, a key regulator of inflammation. The same cellular model was used by Zhou et al. (2018b), who confirmed the anti-neuroinflammatory effect of several phenolic compounds isolated from cannabis seeds, particularly a coumaroyl amino glucoside derivative, significantly reducing TNF-α levels. Caffeoyltyramine has also been shown to have neuroprotective properties in a dopaminergic cell line, providing protection against H_2_O_2_-induced cell death (Maiolo et al. 2018). Using H_2_O_2_-induced PC12 cell damage models, a recent study demonstrated the neuroprotective properties of several lignanamides, namely cannabisin Ⅰ, cannabisin Ⅲ, cannabisin A, cannabisin M, 3,3'-demethylgrossamide, cannabisin E, lyciumamide C, and (7'R)-*N-trans*-cinnamoyloctopamine (Zhang et al. 2024). Moreover, two nor-lignanamides isolated from cannabis seeds ((±)-Sativamides A and B) have demonstrated neuroprotective activity in PC-12 and SH-SY5Y cells subjected to Tm-induced endoplasmic reticulum stress (Zhu et al. 2018). All these studies indicate the potential of phenolic compounds from cannabis seeds in drug discovery for the treatment of neurodegenerative diseases, such as Alzheimer's and Parkinson's.

### Anti-inflammatory activity

Aloo et al. (2023) tested the anti-inflammatory activity of cannabis seeds and stems based on the protein denaturation inhibition method. The seed extract showed stronger inhibition than the stem extract and aspirin used as a positive control, and thus an interesting anti-inflammatory activity.

Other studies have used other more specific tests, such as the measurement of cytokine production, which are often more directly related to the mechanisms of inflammation. In this context, Rea Martinez et al. (2020) demonstrated that defatted hemp seed extracts, particularly the ethyl acetate fraction rich in lignanamides, exhibited potent anti-inflammatory effects by suppressing key inflammatory mediators (cytokines IL-6 and TNF-alpha) and shifting monocyte populations towards a less pro-inflammatory phenotype. This highlights their potential as natural therapeutic agents for inflammation-related disorders.

Some compounds isolated from cannabis seeds have been evaluated for their potential to modulate enzymes linked to inflammation and disease. Bourjot et al. (2016) tested several lignanamides for their ability to inhibit mammalian arginase, an enzyme involved in the urea cycle and linked to various diseases, including inflammatory diseases. At 100 µM, Caffeoyltyramine, cannabisin B, and 3,3′-demethylgrossamide demonstrated significant inhibitory effects, with 62.5, 60.8, and 50.9% respectively, whereas, cannabisin I, M, and F were less active (35.5, 26.0, and 0%, respectively). In another study, Kim et al. (2023) assessed the inhibitory activity of lignanamides isolated from hemp seed hulls against soluble epoxide hydrolase. Cannabisin I, cannabisin A, cannabisin B, *N-trans*-caffeoyltyramine, and grossamide showed significant inhibition of this enzyme; epoxide levels were therefore increased, potentially enhancing their beneficial effects on cardiovascular health, inflammation, and pain modulation. These studies may have direct implications in the formulation of new drugs that control inflammation and the progression of chronic inflammatory diseases.

### Dermo-protective activity

Studies on the dermo protective effect of cannabis seeds remain scarce, with most focus on melanogenesis. Melanin, the natural skin pigment, is produced from tyrosine via the enzyme tyrosinase, whose inhibition reduces pigmentation. Two complementary approaches could be used: in-vitro enzymatic assays with tyrosinase and cellular assays with B16F10 melanoma cells to assess effects on melanin production in a biological system. The first study in this context was conducted by Manosroi et al. (2019), who reported an anti-tyrosinase activity of seed extracts with an IC_50_ of 0.07 mg/mL compared with 0.005 mg/mL for the reference kojic acid. In contrast, Michailidis et al. (2021) observed only limited inhibition (< 20%) event at 500 µg mL⁻^1^ in extracts from cannabis seed paste obtained after oil elimination (Vs. IC₅₀ of kojic acid = 50 µM), highlighting the variability in activity depending on extraction methods and/or seed preparation. More refined fractionation approaches appear more promising: Kim et al. (2021) showed that the ethyl acetate fraction of cannabis seeds exhibited the most significant antityrosinase activity compared to other fractions. Their study further tested the individual effect of 3 synthesized hydroxycinnamic acid amides (*N-trans*-caffeoyltyramine, *N-trans*-feruloyltyramine, and *N-trans-*coumaroyltyramine), and showed that *N-trans*-caffeoyltyramine is the most promising compound, as it effectively reduces melanin in B16F10 melanoma cells and inhibits tyrosinase, without cytotoxicity. In a subsequent study, Kim et al. (2024) identified cannabisins A and B as potent tyrosinase inhibitors, surpassing the effect of kojic acid. Cannabisin A also reduced melanin content and tyrosinase activity in B16F10 melanoma cells. Beyond pigmentation, skin aging is another relevant cosmetic target, yet only one study has investigated this aspect. Michailidis et al. (2021) assessed anti-collagenase and anti-elastase activities of extracts from cannabis seed paste. The extracts showed strong anti-collagenase activity, reaching up to 98.5% inhibition at 600 µg mL⁻^1^ (positive control phosphoramidon IC₅₀ = 16 µM), whereas anti-elastase activity was weaker with inhibition remaining below 40% even at a concentration of 300 µg mL⁻^1^ (positive control elastinal IC₅₀ = 0.5 µg mL⁻^1^). Supporting potential real-world applications, a clinical study demonstrated the positive effect of a 3% cannabis seed extract cream on sebum and erythema of human cheek skin, suggesting its use as a treatment of acne vulgaris, seborrhea, papules and pustules (Ali and Akhtar 2015). These outcomes suggest that compounds derived from cannabis seeds can influence cutaneous lipid regulation and inflammatory processes.

These aforementioned results suggest that hemp seed extracts and their phenolics may be useful in cosmetic formulations, specifically those targeting hyperpigmentation and skin aging. However, challenges such as variability in extraction methods, potential stability issues, limited studies on isolated molecules compared with whole extracts, and the need for rigorous in-vivo and clinical validation must be addressed to confirm their efficacy and safety.

### Antibacterial activity

Several studies have reported antibacterial activity in cannabis seed extracts. Alonso-Esteban et al. (2022) systematically evaluated the activity of cannabis seed extracts from several varieties, at concentrations from 0.005 to 5 mg/mL, against a panel of Gram-positive and Gram-negative bacteria. Their result revealed a complex, strain- and variety-dependent activity profile. Notably, whole seeds of variety “KC Dora” exhibited strong inhibition of *Bacillus cereus*, *Staphylococcus aureus*, *Enterococcus faecalis,* and *Salmonella Typhimurium,* with minimum inhibitory concentrations (MIC = 0.01–0.15 mg/mL) and minimum bactericidal concentrations (MBC = 0.02–0.3 mg/mL) lower than those of the reference antibiotics streptomycin and ampicillin. In contrast, other varieties displayed limited activity; for example, whole seeds of “Fedora 17” were ineffective against *Listeria monocytogenes* and *Escherichia coli* (both with MIC/MBC of 0.9/1.2 mg/mL). In the same context, Frazzini et al. (2024) demonstrated an inhibition of bacterial growth of *Escherichia coli* reaching 87% at 10 mg/mL of hulled cannabis seed extract. However, this evaluation did not include comparisons with standard antibiotics, which constrains the ability to gauge the relative potency and spectrum of the observed antibacterial effects.

A selective antimicrobial activity of *C. sativa* seed extract was recorded against pathogenic strains, namely *Enterobacter aerogenes*, *Salmonella Typhimurium*, *Escherichia coli*, *Staphylococcus aureus*, and *Enterococcus faecalis*, with no inhibitory effect on the growth of probiotic strains belonging to the genera *Bifidobacterium* and *Lactobacillus*. The effective concentrations ranged from 1 to 2.5 mg/mL, indicating moderate potency compared with conventional antibiotics, which are effective at much lower concentrations. Interestingly, *C. sativa* extract (at 0.5 mg/mL) inhibited the biofilm-producing strain *S. aureus* ATCC 35556, suggesting potential applications in human health and biomedical industry (Frassinetti et al. 2020). In an innovative approach, Dobrucka et al. (2025) recently used ethanolic extracts of dehulled seeds to produce films for use as bio-based active food packaging material. Their results showed that all prepared films had inhibitory activity against *S. aureus* and lethal effect against *S. typhimurium* strain (Dobrucka et al. 2025), offering exploitable potential in the food and nutraceutical industry as an alternative to antibiotics and antibacterial compounds.

Nevertheless, it is noteworthy that none of these studies tested isolated phenolic compounds, and the reported activities were observed using whole seed extracts. When phenolic profiling was performed, only a limited number of compounds were detected (Alonso-Esteban et al. 2022; Frazzini et al. 2024), suggesting that additional, uncharacterized phenolics or synergistic interactions may contribute to the observed antimicrobial effects. These limitations underscore the challenges in attributing antibacterial activity to specific phenolic constituents and highlight the need for more comprehensive characterization of extract composition, alongside standardized antimicrobial testing.

### Metabolic regulatory activity

α-amylase and α-glucosidase are two key digestive enzymes that play an important role in carbohydrate metabolism. Inhibition of these enzymes helps slow down starch digestion and glucose absorption, which helps control postprandial blood glucose levels. Lipase, on the other hand, is involved in lipid metabolism and its inhibition helps decrease lipid absorption, which can be useful for weight management and fat metabolism-related problems, such as hyperlipidemia or obesity.

Cannabis seed extract showed significant anti-obesity and antidiabetic activities, exhibiting 75.67% lipase inhibition and 76.14% α-glucosidase inhibition, at 1 mg/mL concentration (Aloo et al. 2023). Similarly, seed cake extracts effectively inhibited α-glucosidase, indicating a potential antidiabetic effect, likely due to *N-trans*-caffeoyltyramine (r = 0.99, p < 0.01) (Leonard et al. 2021a). In addition, El-mernissi et al. (2024) found that cannabis seed extract showed potent in-vitro α-amylase inhibitory activity (IC_50_ = 25.02 μg/mL). In-vivo studies have shown significant favorable effects on alloxan-induced diabetic rats, including reduction in blood glucose and lipids, as well as hepatoprotective and nephroprotective properties (El-mernissi et al. 2024). In another in-vivo study, the effect of cannabis seeds on hindgut function, antioxidant status, and lipid metabolism was investigated in diet-induced obese rats. The results showed that dietary supplementation with a relatively small amount of whole or defatted hemp seeds can partially alleviate high-fat diet-induced disorders in rats, although it is unable to prevent the development of obesity itself (Jurgonski et al. 2020). These studies demonstrate the role of cannabis seed extracts in regulating lipid and carbohydrate metabolism, with potential nutraceutical applications in the management of diabetes and certain metabolic disorders.

Collectively, the available evidence presented in this section demonstrates that phenolic compounds from cannabis seeds exert a broad spectrum of biological activities, spanning antioxidant, anticancer, neuroprotective, anti-inflammatory, dermo-protective, antibacterial, and metabolic regulatory effects. These activities are consistently supported by in-vitro, ex-vivo, and, to a lesser extent, in-vivo models, with phenolic-rich extracts and isolated phenylpropionamides emerging as key bioactive contributors. Antioxidant activity appears as a central and unifying mechanism, closely linked to phenolic content and often underlying other biological effects. However, biological outcomes remain highly context-dependent, influenced by extract composition, processing history, target model, and whether whole extracts or isolated compounds are evaluated. While these findings highlight the strong potential of cannabis seed phenolics for various applications, they also underscore the need for deeper mechanistic investigations, standardized bioassays, and translational studies in real-world applications.

## From bench to market: challenges in translating cannabis seed phenolics into real products

A growing body of literature highlights the phenolic richness and functional potential of cannabis seeds, as discussed in the previous section. However, the translation from laboratory-scale research to commercially viable products remains complex. This translational gap arises from connected scientific, technological, regulatory, and market-related challenges that must be critically addressed to enable successful valorization of cannabis seed phenolics.

One of the primary barriers lies in the extraction and processing strategies. While laboratory-scale studies frequently optimize extraction yields and phenolic richness, scaling these methods to industrial levels introduces new constraints, to balance efficiency, cost, and sustainability. Additionally, fractionation and purification steps intended to enrich bioactive phenolics may inadvertently lead to compound degradation or loss when the processes are not carefully optimized and controlled.

Beyond compositional analysis, the validation of biological activity represents a critical bottleneck in the bench-to-market pathway. Although numerous in-vitro studies report strong functional capacity, these findings do not necessarily translate into physiological efficacy. Plant phenolic compounds could also exhibit limited bioavailability due to poor absorption, extensive metabolism, or rapid elimination (Brglez Mojzer et al. 2016). Moreover, the biological effects observed in complex phenolic matrices may arise from synergistic interactions that are not captured when individual compounds are tested in isolation. The lack of in-vivo/clinical evidence, bioavailability studies, and compound-specific activity, thus restricts the strength of functional claims and limits industrial adoption.

Formulation constitutes another decisive stage in the translational process. Hemp seed phenolics are chemically reactive and may exhibit limited stability when incorporated into food, cosmetic, or nutraceutical matrices. Ensuring adequate shelf life without compromising activity often necessitates protective strategies, such as encapsulation or the use of stabilizing carriers.

In addition, regulatory considerations constitute a major non-scientific hurdle in bringing hemp-seed phenolic products to market. According to the Food and Drug Administration in the United States and the European Food Safety Authority in the European Union, hemp seeds and their traditional derivatives (oil and protein), are recognized as safe for food use, provided that THC levels remain negligible. However, this recognition does not automatically extend to hemp seed crude extracts or phenolic-enriched fractions. Such products are therefore generally subject to additional regulatory scrutiny to ensure the absence of residual cannabinoids and to demonstrate safety at the intended use level. Only fully purified phenolic compounds, free from any THC contamination, can generally be treated like other plant-derived ingredients in foods, supplements, or cosmetics. Regardless of the form, ensuring safety, demonstrating efficacy, and meeting applicable regulatory requirements remain essential for successful product approval and commercialization. Moreover, market-related challenges also shape the translational landscape: Cannabis seed phenolics must compete with well-established natural antioxidants and bioactive compounds, which are often cheaper and supported by extensive clinical data.

In this context, successful translation of cannabis seed phenolics from bench to market requires an integrated and multidisciplinary approach. Coordinated efforts that link extraction science, analytical chemistry, bioavailability studies, and regulatory strategy are essential to overcome existing bottlenecks. By aligning scientific rigor with industrial and consumer expectations, hemp seed phenolics hold significant promise as functional ingredients, provided that translational challenges are addressed in a systematic and evidence-based manner.

## Limitations and inconsistencies

After analysis of the available literature on phenolic compounds from cannabis seeds, several limitations and inconsistencies were identified that should be carefully considered by the scientific community.

First, the analysis of phenolic content and associated biological activities of cannabis seed extracts presents several challenges, especially when comparing results across studies. These difficulties mainly arise from methodological heterogeneity, including differences in extraction procedures, analytical techniques, incomplete sample description, and the use of different units to express results. In some cases, the absence of appropriate positive controls further limits the reliability and comparability of reported biological activities. Moreover, variability in extract composition (often insufficiently characterized at the molecular level) complicates the attribution of observed effects to specific phenolic compounds. Differences in experimental models, such as the cancer cell lines, microbial strains, or bioassays employed, add another layer of complexity to data interpretation. In some studies, biological outcomes are reported primarily as qualitative trends or graphical representations without precise quantitative values, restricting data extraction and synthesis. Additionally, when extract concentrations or exposure conditions are not clearly specified, it becomes difficult to establish dose–response relationships or to assess the true potency of the tested extracts. To overcome these issues, the adoption of common reporting standards is strongly recommended. Such standards should ensure transparent and systematic reporting of key methodological elements, including detailed sample description, extract composition, standardized units of measurement, inclusion of appropriate positive controls, and clearly defined experimental conditions. Improved standardization would greatly enhance the robustness, reproducibility, and interpretability of future studies on cannabis seed phenolics.

Second, some studies include cannabinoid analysis as part of their objective to characterize the composition of cannabis seeds, without mentioning that the presence of cannabinoids in the seeds is primarily due to contamination by resin glands during processing rather than endogenous synthesis. This omission may mislead the reader. Other studies confuse the regulatory limits for cannabis cultivation with those for the commercialization of cannabis-derived products intended for consumption. The regulatory limits for cannabis cultivation always refer to the plant's flowering tops, not the seeds. Cannabinoid analyses are conducted on seeds intended for commercialization to mitigate the risk associated with THC contamination only.

Third, the examination of several studies has revealed that the nomenclature of certain classes of phenolic compounds in cannabis seeds poses challenges or inconsistencies. For instance, several studies use the term "phenylpropionamides" to designate both HCAAs and lignanamides, while others use “phenylpropanamide” or "phenylpropanoids." The latter term is also used to refer to other phenolic compounds (including some phenolic acids and flavonoids), since they all derive from the phenylalanine via the phenylpropanoid metabolic pathway. Moreover, some studies classify phenylpropionamides as alkaloids (Ning et al. 2022). Broadly speaking, alkaloids are defined as plant secondary metabolites containing nitrogen, leading some researchers to extend the term to include non-heterocyclic nitrogenous compounds like phenylpropionamides. They could be categorized as "non-heterocyclic alkaloids" or "phenolic alkaloids." While these differences in naming are acceptable within the scientific community (because the compounds might share similar structures or properties), it’s important for readers to be cautious and carefully examine the definitions in each context when comparing results from various studies.

Finally, some misconceptions were detected. As indicated in the introduction section, cannabis fruit and seed are often confused and referred to by the same term due to a common misuse of terminology. In addition, the terms cake and meal are used interchangeably while the two are distinct. Cake is generally the residue of the oil extraction by mechanical pressing, while meal is the residue after complete defatting, generally using an organic solvent. Similarly, the term “flour” could refer to the obtained powder by grinding either the cake or meal, and in some cases, it refers to the powder produced by grinding the seeds directly. These subtle differences can significantly impact the final reported phenolic content, since cannabis seeds are rich in lipids (up to 35%). It is important for the reader to consider these distinctions when interpreting or comparing the results.

## Conclusion and future directions

This review has provided an overview of current research on cannabis seeds, with a particular focus on phenolic compounds. After presenting and discussing the available knowledge and advances made regarding their composition, extraction, isolation, and identification methods, as well as their biological activities, some gaps and aspects that remain to be explored have been highlighted. Future research should focus on integrating green extraction techniques to ensure environmental sustainability, safety, and efficiency in extracting phenolic compounds from cannabis seeds. In addition, more interest should be given to optimize extraction and analysis methods to efficiently isolate these compounds, identify the specific phenolics responsible for the observed biological effects, and better understand their bioavailability. In addition, further studies will be needed to confirm their therapeutic effects in in-vivo models, as well as to assess their long-term safety. Further studies will also be needed to explore the mechanisms of action of phenolic compounds and their interaction and synergistic effects with other bioactive components. Finally, scarce data are available regarding the potential of cannabis seed phenolic compounds as dermato-cosmetic agents, concretizing an opportunity for future research in this area. Indeed, the development of new industrial applications, such as cosmetic products enriched with cannabis seed extracts, could play a crucial role in expanding their large-scale use. In summary, cannabis seeds represent a valuable resource but still largely underexploited. As research progresses, cannabis seeds could offer innovative solutions in various fields, thus meeting a growing need for natural and bioactive ingredients in modern industry.

## Data Availability

All data discussed in this review are available from previously published studies, which are cited throughout the manuscript. No primary research results or new datasets were generated.

## Abbreviations

4CL: 4-Hydroxycinnamoyl-CoA ligase
ABTS: 2′-Azino-bis(3-ethylbenzothiazoline-6-sulfonic acid)
AChE: Acetylcholinesterase
C3H: *p*-Coumarate 3-hydroxylase
C4H: Cinnamate 4-hydroxylase
CAA-RBC: Cellular antioxidant activity in Red Blood Cells
CAT: Catalase
CD: 2-Hydroxypropyl-β-cyclodextrin
CHS: Chalcone synthase
COMT: Caffeic acid O-methyltransferase
CUPRAC: Cupric Ion Reducing Antioxidant Capacity
DNA: Deoxyribonucleic Acid
DPPH: 2,2-Diphenyl-1-picrylhydrazyl
DW: Dry Weight
EAE: Enzyme-assisted extraction
ESI–MS: Electrospray Ionization Mass Spectrometry
FRAP: Ferric reducing antioxidant power
FTIR: Fourier Transform Infrared (Spectroscopy)
GAE: Gallic Acid Equivalents
GI_50_: Half-maximal growth inhibition concentration
GPx: Glutathione peroxidase
HCAA: Hydroxycinnamic acid amides
HPLC: High Performance Liquid Chromatography
HR-MS: High-resolution mass spectroscopy
IC_50_: Half-maximal Inhibitory Concentration
IL: Interleukin
LDH: Lactate dehydrogenase
LDL: Low-Density Lipoprotein
LPS: Lipopolysaccharide
LUE: Luteolin Equivalents
MAE: Microwave-assisted extraction
MBC: Minimum bactericidal concentration
MIC: Minimum inhibitory concentration
MS: Mass spectroscopy
NF-κB: Nuclear Factor kappa-light-chain-enhancer of activated B cells
NMR: Nuclear magnetic resonance
ORAC: Oxygen Radical Absorbance Capacity
PAL: Phenylalanine ammonia-lyase
PDA/DAD: Photodiode Array Detector / Diode Array Detector
PEF: Pulsed Electric Field
prep-HPLC: Preparative high performance liquid chromatography
QE: Quercetine equivalent
QTOF: Quadrupole-Time-of-Flight
ROS: Reactive oxygen species
RP-CC: Reversed-phase column chromatography
RSM: Response Surface Methodology
SEC: Size exclusion chromatography
SFE: Supercritical fluid extraction
SiO_2_-CC: Silica gel column chromatography
SOD: Superoxide dismutase
TAC: Total Antioxidant Capacity
TBARS: Thiobarbituric Acid Reactive Substances
TE: Trolox equivalent
TFC: Total flavonoid Content
THC: Δ⁹-Tetrahydrocannabinol
TLC: Thin layer chromatography
TNF: Tumor necrosis factor
TOF: Time-of-Flight
TPC: Total Phenolic Content
UAE: Ultrasound-assisted extraction
UHPLC: Ultra-High-Performance Liquid Chromatography
QQQ-MS/MS: Triple Quadrupole Tandem Mass Spectrometry
UV: Ultraviolet
